# A Novel hepatocellular carcinoma specific hypoxic related signature for predicting prognosis and therapeutic responses

**DOI:** 10.3389/fimmu.2022.997316

**Published:** 2022-08-17

**Authors:** Guangzhen Cai, Jinghan Zhu, Deng Ning, Ganxun Li, Yuxin Zhang, Yixiao Xiong, Junnan Liang, Chengpeng Yu, Xiaoping Chen, Huifang Liang, Zeyang Ding

**Affiliations:** Hepatic Surgery Center, and Hubei Key Laboratory of Hepatic-Biliary-Pancreatic Diseases, Tongji Hospital, Tongji Medical College, Huazhong University of Science and Technology, Wuhan, China

**Keywords:** hypoxia, prognosis, tumor microenvironment, drug sensitivity, hepatocellular carcinoma

## Abstract

Hypoxia is an important feature of the tumor microenvironment(TME) and is closely associated with cancer metastasis, immune evasion, and drug resistance. However, the precise role of hypoxia in hepatocellular carcinoma(HCC), as well as its influence on the TME, and drug sensitivity remains unclear. We found the excellent survival prediction value of Hypoxia_DEGs_Score model. In hypoxic HCC, somatic mutation, copy number variation, and DNA methylation were closely related to hypoxic changes and affected tumorigenesis, progression, metastasis, and drug resistance. In HCC, aggravated hypoxic stress was found to be accompanied by an immune exclusion phenotype and increased infiltration of immunosuppressive cells. In the validation cohort, patients with high Hypoxia_DEGs_Score were found to have worse immunotherapeutic outcomes and prognoses, and may benefit from drugs against cell cycle signaling pathways rather than those inhibiting the PI3K/mTOR pathway. Hypoxia_DEGs_Score has an excellent predictive capability of changes in the TME, the efficacy of immunotherapy, and the response of drugs. Therefore, Hypoxia_DEGs_Score can help develop personalized immunotherapy regimens and improve the prognosis of HCC patients.

## Introduction

Hepatocellular carcinoma(HCC), an aggressive malignancy with a dismal prognosis, is the fourth most common cause of cancer-related deaths worldwide (1). Although the emerging immunotherapy and targeted therapy approaches offer certain benefits, the patients who benefit still account for only a small subset of the total patient population (2, 3). Even if patients have similar clinicopathological characteristics and treatment options, they may still have a completely different prognosis because of the heterogeneity of HCC (4). Therefore, it is imperative to find a signature with high predictive value to guide treatment and improve the prognosis.

Hypoxia is a status of imbalance between the tumor’s oxygen demand and the circulating oxygen supply, mainly because the growth rate of blood vessels cannot keep up with the oxygen demand of the tumor (5–7). To adapt to this status, the tumor will derive a new adaptive dynamic balance to ensure its survival and development (8). Surprisingly, such extreme conditions stimulate the formation of blood vessels, promote invasion and metastasis, increase the instability of the genome, and impair the antitumoral immune response (7, 9). Hypoxia is often particularly obvious and common in advanced solid tumors and is highly correlated with rapid tumor progression, drug resistance, and poor prognosis (8).

The changes in the tumor microenvironment caused by hypoxia involve multiple levels. Hypoxia activates the AKT and ERK pathways through different mechanisms to induce the epithelial-to-mesenchymal transition (EMT) and expression of matrix metalloproteinases (MMPs), which promote the invasion and metastasis of HCC (10). In patients with HCC, the resistance of sorafenib is considered to be related to the hypoxia caused by its anti-angiogenetic effect (11). After reviewing recent research on the characteristics of hypoxia across different cancer types (12–15), we selected 15 genes in the study of Buffa et al. to represent the characteristic genes of hypoxia (12). It is well known that hypoxic stress can drive changes in multiple biological pathways, playing a key role in the occurrence and development of HCC.

Although there have been many advances in HCC and hypoxia research, there are still certain difficulties in the evaluation of the hypoxic status. Direct and accurate measurement of HCC hypoxic status and oxygen amount is still difficult to implement, and hypoxia-inducible factor-1α(HIF-1α) expression cannot accurately reflect all the phenotypes of hypoxia (16). Therefore, it is crucial to find a robust signature that reflects the hypoxic status of HCC to guide treatment decisions.

This study used four HCC datasets for research, encompassing the TCGA-LIHC, ICGC-LIRI-JP, Fudan Zhongshan, and Tongji cohorts, and investigated their hypoxia status. We found that hypoxia was not only correlated with immune cell infiltration, but with genetic instability and epigenetic modifications in HCC. We then constructed a hypoxia score model based on significantly different genes to quantify the hypoxic stress of each sample, which enabled us to assess hypoxia-related changes of tumor biology. Finally, we validated the survival prediction value of Hypoxia_DEGs_Score and confirmed its ability to guide targeted therapy and immunotherapy.

## Materials and methods

### Dataset collection and preprocessing

The workflow diagram of our study is shown in **Figure S1A**. The RNA-seq and clinicopathological data of HCC samples were obtained from four different institutions: TCGA (17), ICGC (18), Zhongshan Hospital (19), and Tongji Hospital(this study). The cohorts of immunotherapy interventions were further included to evaluate the relationship between the Hypoxia_DEGs_Score and the benefits of immunotherapy. The patients in the IMvigor210 cohort had advanced urinary tract transitional cell carcinoma and had received anti-PD-L1 therapy (20). GSE78220 is a dataset of an interventional regimen in metastatic melanoma with anti-PD-1 antibodies (21). The basic information of the datasets is summarized in **Table S1**.

### Unsupervised clustering for 15 characteristic hypoxia-related genes

A total of 15 characteristic hypoxia-related genes (ACOT7, ADM, ALDOA, CDKN3, ENO1, LDHA, MIF, MRPS17, NDRG1, P4HA1, PGAM1, SLC2A1, TPI1, TUBB6, and VEGFA) were selected based on the exploration of gene function and co-expression patterns *in vivo*, after cross-comparison of signature studies related to hypoxic stress (12–15). Unsupervised clustering analysis was used to distinguish the HCC samples according to their hypoxic status. The consensus clustering algorithm in the ConsensusClusterPlus package was used for unsupervised clustering (22, 23).

### Gene set variation analysis

To evaluate signature level discrepancies between different hypoxic statuses and states of diverse functions in individual HCC samples, we applied the GSVA package to assess the differences in various functional gene sets, including Kyoto Encyclopedia of Genes and Genomes (KEGG), Gene ontology(GO), HALLMARK, and the 15 hypoxia hallmark genes (24).

### Evaluation of immune function status and immune cell infiltration

The TIDE algorithm was used to evaluate the immune function status (25). The CIBERSORT algorithm and the ssGSEA algorithm were used to quantify the abundance of infiltrating immune cell (26–28). Immune cell infiltration signatures from the study of Charoentong et al. were used to estimate the infiltration of myeloid-derived suppressor cells(MDSCs) in ssGSEA (26, 27).

### Constructing the Hypoxia_DEGs_Score scoring system to evaluate individual HCC cases

To identify hypoxia-related differentially expressed genes(DEGs) in HCC, we applied the Wilcoxon rank-sum test to determine whether there was a difference in gene expression between the two statuses. The criteria FDR < 0.05, absolute fold change > 1.5, and the same direction of change among the different hypoxia statuses based on TCGA-LIHC and ICGC-LIRI-JP cohorts were used to identify the DEGs. A univariate regression model was used to analyze the hazard ratio of each DEG separately. Then, based on the survival risk of DEGs, a part was selected to be used to construct a scoring system. Based on the univariate survival regression analysis, we defined the Hypoxia_DEGs_Score of each patient through a method analogous to Gene expression grade index (GGI):


$$Hypoxia\_DEGs\_Score=\sum_{i=1}^{n}\left(beta_{i}\times Exp_{i}\right),$$

where i is the selected hypoxia-related DEGs, and beta is the prognostic value of each gene signature score (29). The enrichment analysis and functional annotation of DEGs was conducted using the clusterProfiler package (30).

### Analysis of tumor-associated somatic mutations and CNVs

Mutational landscapes of tumors with different hypoxia statuses were exhibited in waterfall plots using the maftools package (31). The tumor-associated mutated genes demonstrated in the waterfall plot were downloaded from the cBioPortal website (32). We performed enrichment analysis for their corresponding genes for the 40 differential CNV loci that satisfy FDR< 0.05 for differential expression, and the expression changes were consistent with the changes of CNV.

### Changes of DNA methylation

The analysis of DNA methylation differences in tumors with different hypoxia status was performed using the ChAMP package (33). Before analysis, the low-quality probes were removed and the missing values were imputed. The transcriptional repression caused by DNA methylation is mainly due to CpG methylation in the promoter region, after which the binding of CPG-binding proteins in the promoter region obstructs the binding of transcription factors to the promoter (34). Therefore, we selected candidate sites based on fluctuations in the beta value of individual promoter CpG islands. Differentially methylated positions (DMPs) were selected within 2000 bases upstream of the transcription start site, based on the criteria FDR< 0.01 and |delta beta| >0.15.

### Correlation of the Hypoxia_DEGs_Score with drug response

We obtained transcriptome data of 1000 cancer cell lines and the drug response data from Genomics of Drug Sensitivity in Cancer (GDSC) (35). To further analyze the association between hypoxia and drug sensitivity in HCC, we used the pRRophetic package to predict drug effects in HCC patients (36). The transcriptome data of 25 HCC cell lines from GDSC were merged with HCC transcriptome data from TCGA-LIHC after batch effects were removed using the SVA package (37), followed by principal component analysis (PCA) to assess the hypoxic status of different HCC cell lines.

### Cell proliferation assay

The HCC cell lines Huh7 and HepG2 were obtained from the China Center for Type Culture Collection (Wuhan, China). Both cell lines were cultured in Dulbecco’s Modified Eagle Medium (HyClone, USA) supplemented with 10% fetal bovine serum (Gibco). HCC cell lines were seeded into 96-well plates at 4000 cells per well(4 replicates). On the 4next day, the medium in the wells was replaced after diluting the drug in equal proportions and placed into a normoxic incubator (37°C, 5% CO2, and 21% O2) or anoxic incubator (37°C, 5% CO2 and 1% O2). AKT_inhibitor_VIII, FH535, BI_2536, and RO_3306 were purchased from MedChemExpress(USA). After 72 hours, the medium was changed to 10% Cell Counting Kit 8 solution and incubated for one hour, after which the absorbance at 450 nm was recorded using an ELx 800 Universal Microplate Reader(BIO-TEK, USA).

### Protein–protein interaction network of hypoxia-related DEGs

STRING was used to evaluate the associations between 251 hypoxia-related DEGs (38), and the PPI network was visualized using Cytoscape (39). The degree method in the CytoHubba package was used to distinguish the hub-genes among 251 hypoxia-related DEGs, with a threshold of 12 (40).

### RNA-sequencing data from the Tongji Hospital cohort

Tumor samples were obtained from 30 HCC patients. All patients underwent one-stage radical resection in Tongji Hospital from May 2015 to November 2015, without prior anticancer treatment. This study was approved by the Research Ethics Committee of Tongji Hospital, and all patients provided written informed consent forms. RNA library construction and sequencing (**Tables S8** and **S9**) were performed by Novogene Corporation (China).

## Results

### Hypoxic status in HCC stratified based on the hypoxia signature

**Figure 1:**
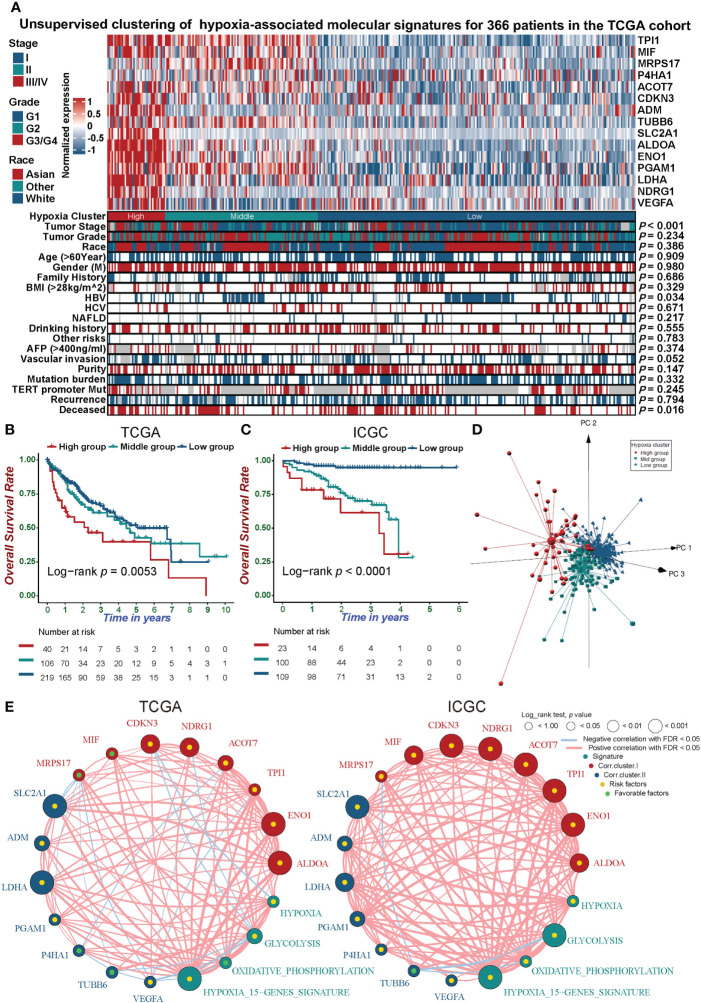
Unsupervised clustering analysis distinguishes hypoxic phenotypes of HCC. **(A)** Unsupervised clustering analysis of the 15-gene hypoxia signature with a comparison of clinicopathological features between three distinct clusters for TCGA-LIHC (ICGC-LIRIJP in **Figure S2A**). **(B, C)** Kaplan-Meier curves demonstrated that hypoxia phenotypes were highly associated with the overall survival of 366 patients in the TCGA-LIHC cohort **(B)** and 232 patients in the ICGC-LIRI-JP cohort **(C)**. **(D)** Principal component analysis for the expression profiles of three hypoxia clusters in the TCGA-LIHC cohort exhibiting a remarkable difference in expression between different hypoxia clusters. **(E)** The interplay between 15 hypoxia signature genes and hypoxia-related signatures in hepatocellular carcinoma. Green dots in the circle, favorable prognostic factors; yellow dots in the circle, risk factors. The lines linking the signature genes indicate their interplay, and thickness reflects the strength of correlation between the signature genes.

We chose a 15-gene hypoxia signature, which exhibited the best robustness in identifying hypoxic status in various cancers in recent studies (12–14), to distinguish the hypoxic status of HCC. The unsupervised clustering of TCGA-LIHC and ICGC-LIRI-JP cohorts showed that the hypoxia status could be distinguished among different HCC samples using the 15-gene hypoxia signature (**Figures 1A** and **S2A**). Further PCA of the clustering results showed that the hypoxia feature in the three-dimensional plot also distinguishes the different hypoxia statuses of the HCC samples (**Figure 1D**). The high hypoxia group showed the shortest overall survival in the TCGA-LIHC and ICGC-LIRI-JP cohorts (**Figures 1B, C**). We found that the 15 genes exhibited significant differences in expression between the adjacent tissue and different groups of tumor samples (**Figures S2B, C**). The hypoxia signature network delineated a comprehensive landscape of hypoxia signature genes, hypoxia-related pathways, and prognostic factors (**Figures 1E** and **S3A**). We found a significant correlation between the expression of 15 genes in the hypoxia signature and a positive correlation between the enrichment scores (ES) of the 15 gene hypoxia signature with hallmark_hypoxia. In the TCGA-LIHC cohort, CDKN3 and TUBB6 had two missense mutations, while NDRG1 and P4HA1 had one missense mutation (**Figure S3B**). We explored the association between CNV and the expression of 15 signature genes, and found that ENO1, TPI1, ACOT7, NDRG1, MIF, ADM, PGAM1, P4HA1, and VEGFA were positively correlated with the high expression of the hypoxia-related genes (**Figure S3C**). These results indicated that the 15-gene hypoxia signature could distinguish different hypoxia statuses of HCC samples at the transcriptomic level.

### Distinct hypoxia clusters associated with hypoxia-related biological processes and the TME

**Figure 2:**
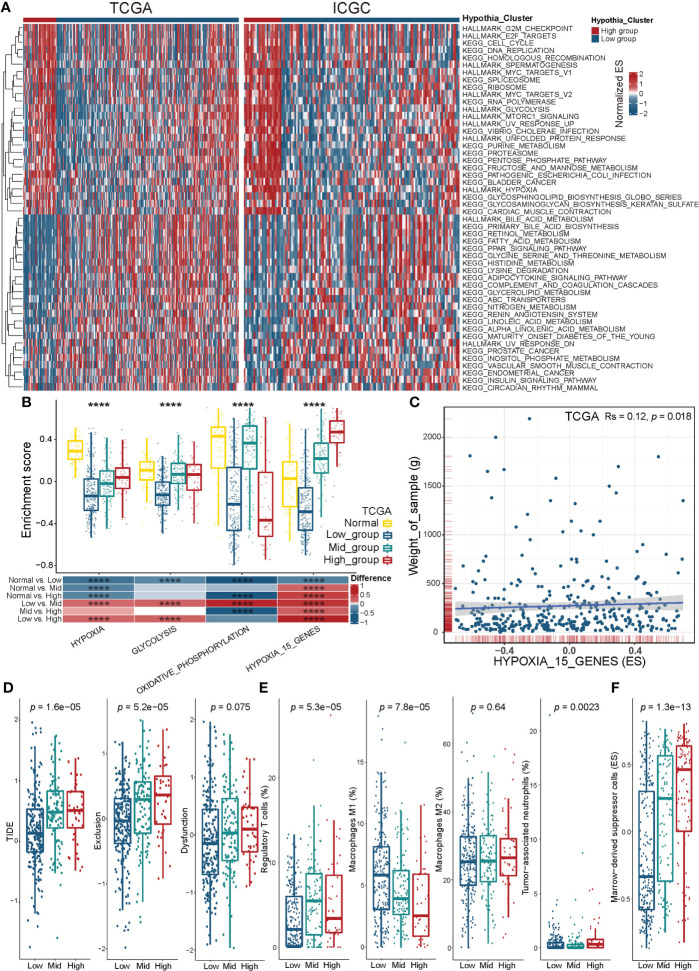
Biological characteristics and tumor immune microenvironment of distinct hypoxia groups. **(A)** Heatmap visualizing the GSVA enrichment analysis based on KEGG and HALLMARK terms showing the activation states of biological pathways in hypoxia high and low groups. Red, activated pathways; blue, inhibited pathways. KEGG, Kyoto Encyclopedia of Genes and Genomes (GO in **Figure S4A**). **(B)** Enrichment score of hypoxia-related pathways across distinct hypoxia groups and normal tissue group in the TCGA-LIHC cohort. Upper panel, boxplot showing the ES distribution and overall variance. Bottom panel, heatmap showed between-group differences for pairwise comparisons. P < 0.05 in the Kruskal-Wallis (upper panel) and Wilcoxon tests (bottom panel) was considered statistically significant. ****P < 0.0001. **(C)** Scatter plots showing the significant association between the hypoxia-related 15-gene signature (ES) and tumor weight according to Spearman’s rank correlation analysis in the TCGA-LIHC cohort. Rs is the coefficient of rank correlation. **(D)** Box plots showing differences in the TIDE, Exclusion, and Disfunction scores between distinct hypoxia groups in the TCGALIHC cohort. **(E, F)** Box plots showing differences in the infiltration of Tregs, M1 macrophages, M2 macrophages, TANs **(E)**, and MDSCs **(F)** between distinct hypoxia groups in the TCGA-LIHC cohort. The infiltration of Tregs, M1 macrophages, M2 macrophages, TANs, and MDSCs was estimated using CIBERSORT **(E)** and ssGSEA **(F)**, respectively. The Kruskal-Wallis test was used to determine the statistical significance of the difference.

To compare the biological behavior of tumors from high and low hypoxia clusters, we performed GSVA enrichment analysis of Hallmark and KEGG gene sets in the TCGALIHC and ICGC-LIRI-JP cohorts (**Figure 2A**, **Table S2**). Under hypoxia, we found that the glucose, fatty acid, and amino acid metabolism was upregulated; upregulated MYC targets and mTORC1 signaling pathway enhanced proliferation; downregulated PPAR promoted tumor migration (41). In the GO term-based enrichment analysis (**Figure S4A**, **Table S3**), we found that upregulation of the Rho pathway changed the cell morphology under hypoxia, thereby promoting the expansion and metastasis of HCC (42). Hypoxia promoted HCC metastasis through the WNT signaling pathway (43). Hypoxia also inhibited the differentiation of immature B cells, while also downregulating complement activation and apoptotic signaling.

We applied GSVA enrichment scoring to understand the glycolysis and oxidative phosphorylation changes in HCC tumors from different hypoxia groups (**Figure 2B**). The change trend of hypoxia signature ES from Hallmark gene sets was consistent with that of the hypoxia clustering results. We scored ES for the 15-gene hypoxia signature and found that the results were in excellent agreement with the hypoxia cluster groupings. The ES of the hypoxia-related 15-gene set was significantly positively correlated with tumor weight (**Figure 2C**).

We analyzed the differences of TME between the different hypoxia statuses based on TIDE scoring (**Figure 2D**, **Table S5**). TIDE and Exclusion scores increased significantly with higher hypoxia levels. Analysis of tumor infiltration by immunosuppressive cells showed an increase of infiltration by regulatory T cells (Tregs), tumor-associated neutrophils (TANs), and MDSCs, which was correlated with aggravation of hypoxia. The increased infiltration of M1 tumor-associated macrophages(TAM) was meaningfully associated with a reduction of hypoxia (**Figures 2E, F**, and **S4B**).

### Construction of a specific hypoxic signature of HCC

**Figure 3:**
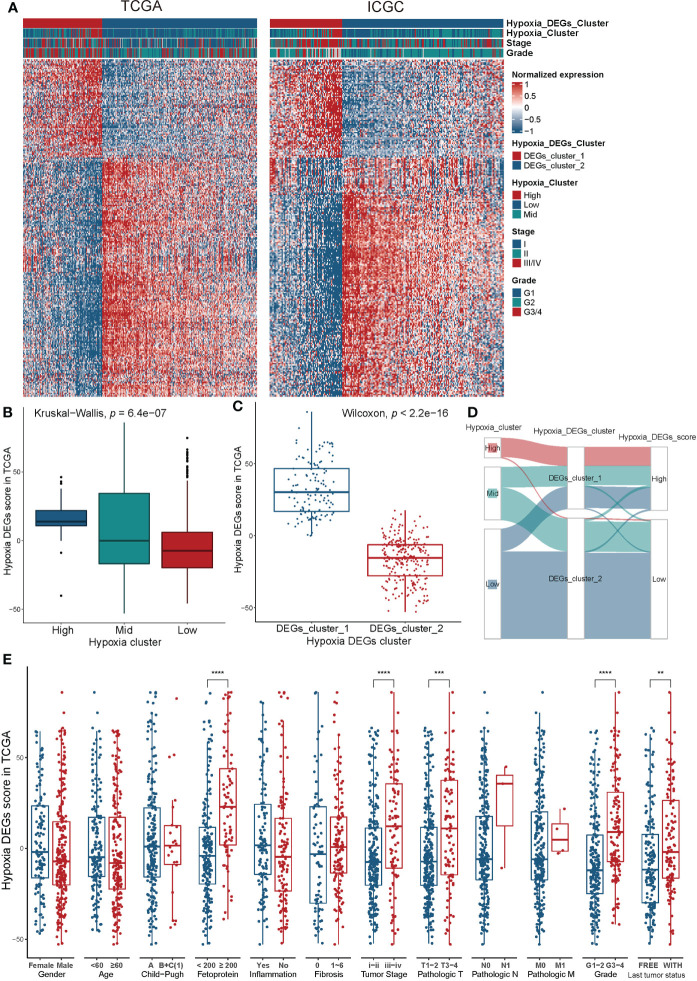
Construction of an HCC-specific hypoxia signature. **(A)** Unsupervised clustering of the hypoxia-related differentially expressed genes in the TCGA-LIHC and ICGC-LIRI-JP cohorts. Red, high expression; blue, low expression. **(B, C)** Differences in the Hypoxia_DEGs_Score between hypoxia clustered groups **(B)** and hypoxia DEG clusters **(C)** in the TCGA-LIHC cohort. **(D)** Sankey diagram exhibiting the shifts of 15-gene hypoxia signature clusters, hypoxia DEG clusters, and Hypoxia_DEGs_Score groups based on the TCGA-LIHC cohort. The distinction between high and low Hypoxia_DEGs_Score groups is based on the optimal cutoff value of the survival analysis. **(E)** Differences in the Hypoxia_DEGs_Score between various clinicopathological features. The Wilcoxon test was used to determine the statistical significance of the differences. **P < 0.01, ***P < 0.001, **** P < 0.0001.

To further explore the changes brought about by hypoxia in HCC, we identified 251 differentially expressed signature genes (**Table S4**). Considering the complexity and breadth of the impact of hypoxia on tumor biology, we quantified the hypoxia signature of individual HCC patients based on a scoring model of these DEGs, called Hypoxia_DEGs_Score(Hypoxia Differential Expression Gene Score; see methods). We performed an unsupervised clustering analysis based on the expression of 251 DEGs. This analysis divided patients with different hypoxia statuses into Hypoxia_DEGs_Cluster_1 and Hypoxia_DEGs_Cluster_2 (**Figure 3A**). We evaluated the Hypoxia_DEGs_Score of the high, mid, and low hypoxia groups. The Hypoxia_DEGs_Score in the hypoxia group also showed an increasing trend (**Figure 3B**). The Hypoxia_DEGs_Score of Cluster_1 was remarkably higher than that of Cluster_2 (**Figure 3C**). In the Sankey diagram, the high and low grouping results of Hypoxia_DEGs_Score were highly consistent with the results of the hypoxia-related DEG clustering (**Figure 3D**, **Table S7**). And Hypoxia_DEGs_Score positively correlated with the transcript level of HIF1A (**Figures S4C, D**).

**Figure 4:**
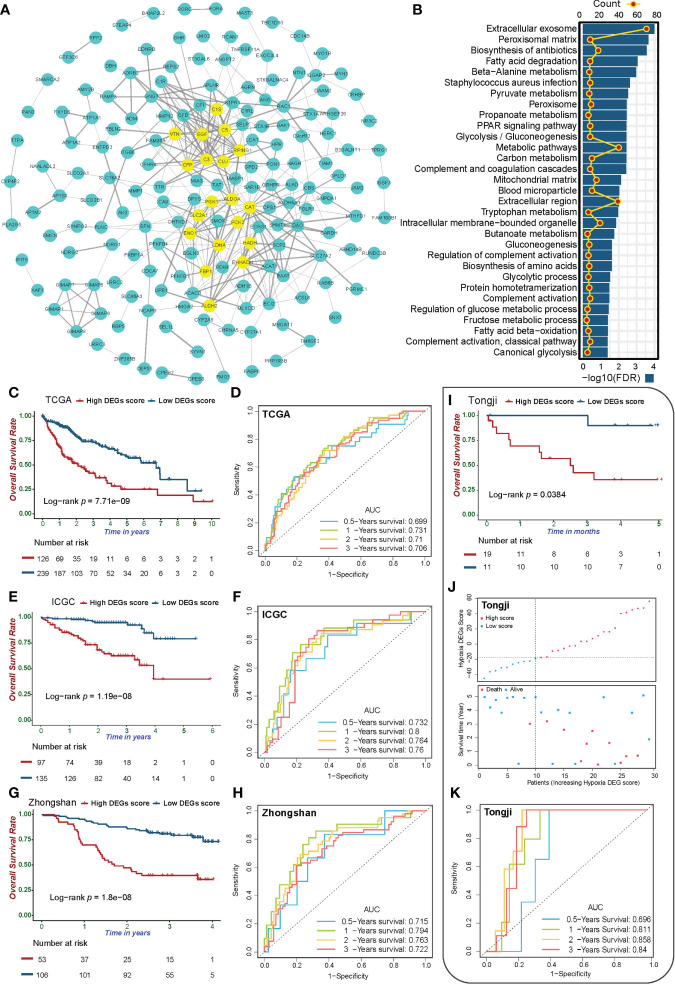
Validation of the Hypoxia_DEGs_Score survival prediction value. **(A)** Protein-protein interaction network of the hypoxia-related DEGs. The thickness of the lines represents the strength of the association, yellow circles represent the hub gene in the PPI network, and blue represents non-hub genes. DEGs with a Degree value greater than the threshold of 12 were considered hub genes. **(B)** Enrichment analysis for the 251 hypoxiarelated DEGs based on KEGG and GO term analysis. **(C, E, G, I)** Kaplan-Meier survival curves showing differences in overall survival between high and low Hypoxia_DEGs_Score groups in the TCGA-LIHC **(C)**, ICGC-LIRI-JP **(E)**, Zhongshan **(G)**, and Tongji **(I)** cohorts. **(D, F, H, K)** ROC analysis of 0.5-, 1-,2- and 3-year OS demonstrating the outstanding prognostic value of Hypoxia_DEGs_Score in the TCGALIHC **(D)**, ICGC-LIRI-JP **(F)**, Zhongshan **(H)**, and Tongji **(K)** cohorts. **(J)** Scatterplots of survival status and risk curves sowing the prognosis and Hypoxia_DEGs_Score values of patients in the Tongji cohort.

We analyzed the 251 DEGs using the STRING database, and identified 19 hub genes with values greater than the threshold of 12 (**Figure 4A**). The enrichment analysis of 251 hypoxia DEGs found that the main effects of the signature of hypoxia-related DEGs were still in energy metabolism-related pathways (**Figure 4B**). In addition, secretion of exosomes, complement activation, and PPAR signaling were also found to be involved in the biological effects of hypoxia in HCC.

### Clinicopathological characteristics associated with the Hypoxia_DEGs_Score

We compared the changes of Hypoxia_DEGs_Score stratified by different pathological features. A high Hypoxia_DEGs_Score was found in patients with elevated alpha-fetoprotein, higher tumor stage, worse pathological stage and grade, as well as a higher tumor recurrence rate (**Figure 3E**).

Initially, we analyzed the survival predicted capability of the Hypoxia_DEGs_Score model in the TCGA-LIHC and ICGC-LIRI-JP cohorts. Patients with high Hypoxia_DEGs_Score had a significantly poorer prognosis in both cohorts (**Figures 4C, E**, **S5, B**). Hypoxia_DEGs_Score also had excellent survival prediction capability in both cohorts in the ROC analysis (**Figures 4D, F**). Since the TCGA-LIHC and ICGCLIRI-JP cohorts were equivalent to the experimental dataset in our study, the Zhongshan cohort and our cohort from Tongji Hospital were selected as the validation datasets. Further analysis showed that the Hypoxia_DEGs_Score model also had an outstanding capability to predict the clinical outcomes in the Zhongshan and Tongji cohorts (**Figures 4G–K** and **S5C**). Therefore, the Hypoxia_DEGs_Score model exhibited excellent prognostic robustness among the results from four different cohorts from independent sources.

In multivariate Cox regression analysis, Hypoxia_DEGs_Score was also a strong independent predictor of clinical outcomes in both cohorts (**Figure S5D**). A nomogram was constructed for readers to predict the survival probability (**Figure S5E**). The 2- and 3-year calibration curves of the nomogram showed a high consistency between predicted survival probability and actual observation (**Figures S6A, B**).

### Somatic mutations and CNVs under distinct hypoxic circumstances

**Figure 5:**
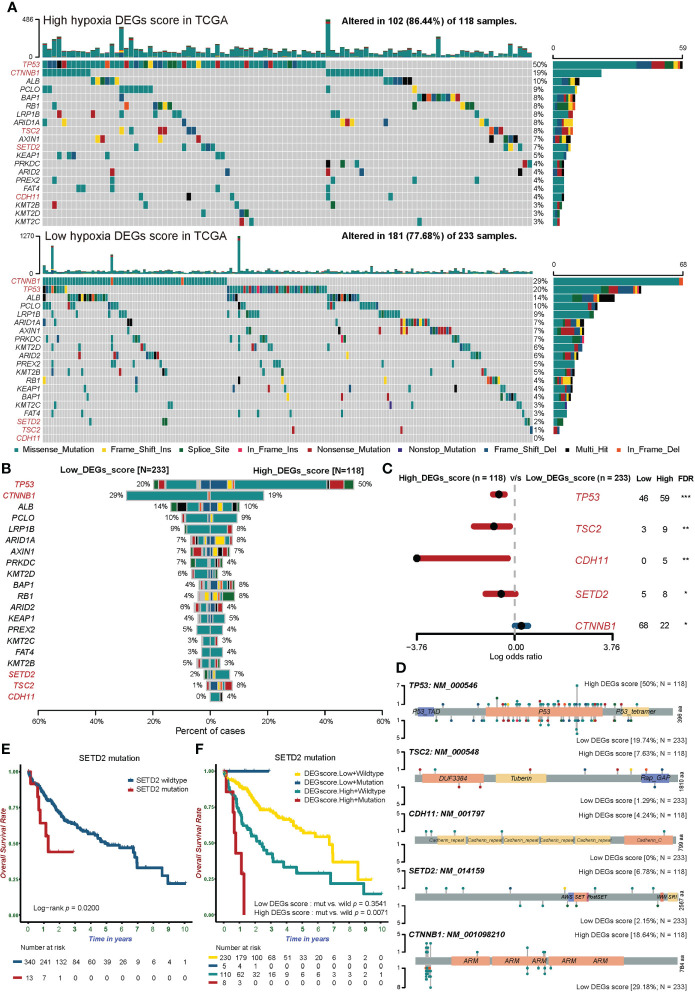
The somatic mutation landscape of tumor-associated genes in groups with different hypoxic statuses. **(A)** Waterfall plots showing the distribution of mutation among the top 20 most frequently mutated tumor-associated genes in the two Hypoxia_DEGs_Score groups from the TCGA-LIHC cohort. Each column represents an individual patient. The upper bar graph shows TMB; the number on the right indicates the mutation frequency in each tumor-associated mutated gene. The right bar graph shows the proportion of each variant type. Mutation types are indicated in the legend at the bottom. **(B)** The bar plot compares mutation rates for tumor-associated mutated genes in the two Hypoxia_DEGs_Score groups. **(C)** Forest plot showing the top 5 most significantly differentially mutated genes between the two groups. The Fisher test was used to determine the statistical significance of the differences. P values were corrected using the Benjamini-Hochberg method. *FDR < 0.05. **FDR < 0.01. ***FDR < 0.001 **(D)** Lollipop plots showing the amino acid residues corresponding to the mutated sites of the top 5 mutated genes. **(E)** Kaplan-Meier survival curves showing differences in overall survival between patients with wild-type or mutant SETD2 in the TCGA-LIHC cohort. **(F)** Kaplan-Meier survival curves demonstrating distinctions in overall survival stratified by both Hypoxia_DEGs_Score grouping and SETD2 mutation status in the TCGA-LIHC cohort. Mut, mutant SETD2; WT, wild-type SETD2.

Somatic mutations not only contribute to the occurrence and development of tumors, but can also change the state of various biological functions of cells (44). We analyzed the mutational profiles and found that mutation probability was lower in the low Hypoxia_DEGs_Score group (**Figure 5A**).

Further analysis showed that the mutation frequencies of tumor suppressor genes (TP53, TSC2, CDH11, and SETD2) in the high Hypoxia_DEGs_Score group representing severe hypoxia were much higher than in the low-score group. At the same time, the mutation frequency of the proto-oncogene CTNNB1 was lower in the high-score group than in the low-score group (**Figures 5B, C**). Mutations of TP53 lead to the rapid proliferation of HCC and promote the progression toward hypoxia (45). Similarly, TSC2 mutations weaken mTOR regulation, leading to tumor cell proliferation and hypoxia (46). The loss of SETD2 is involved in the occurrence and development of HCC (47). The loss of CDH11 function will change the adhesion status resulting in the invasion and metastasis of HCC (48). The profiles of TP53, TSC2, CDH11, SETD2, and CTNNB1 mutation sites are shown in **Figure 5D**.

In further exploration, patients with SETD2 mutations had a significantly worse prognosis (**Figure 5E**). Notably, SETD2 gene mutations accompanied by a high hypoxia status suggested a very poor prognosis. By contrast, there was no difference in the prognosis of patients bearing tumors with mutated and wild-type SETD2 in the low hypoxia group (**Figure 5F**).

**Figure 6:**
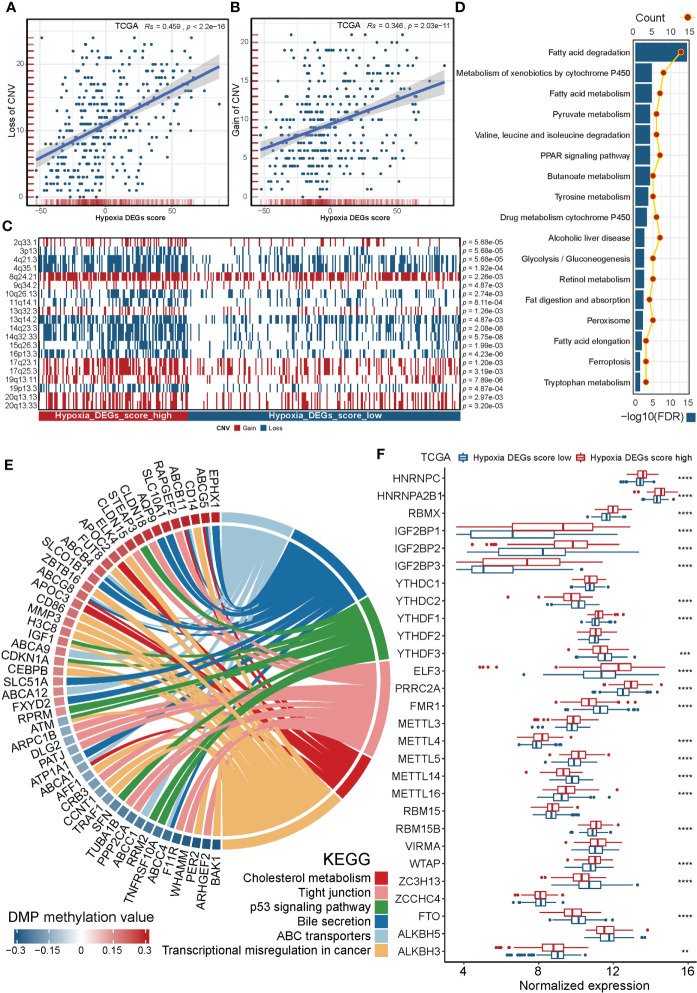
Copy number variation, DNA methylation and N6-methyl-adenosine modification in groups with different hypoxic statuses. **(A, B)** Scatterplots showing the robust correlation of the Hypoxia_DEGs_Score with loss **(A)** and gain **(B)** CNVs according to Spearman’s rank correlation analysis. Rs is Spearman’s coefficient of rank correlation. **(C)** A heatmap exhibiting the top 20 most significantly different CNV loci in distinct Hypoxia_DEGs_Score groups. The Fisher test was used to determine the statistical significance of the differences. Red, deletions; blue, gene number amplifications. **(D)** Enrichment analysis for the associated genes of 40 significantly different CNV loci based on KEGG gene sets. **(E)** Circle diagram showing the enrichment analysis for the targeted genes of DMPs based on KEGG gene sets. FDR< 0.05 for enriched pathways was considered statistically significant and was shown. Red, increased level of DNA methylation in Hypoxia_DEGs_Score high group vs. low group; Blue, decreased level of DNA methylation. **(F)** Boxplots showing the expression of 28 N6-methyl-adenosine regulators in groups with different hypoxic statuses. The boxes indicate the median ± 1 quartile, with the whiskers extending from the hinge to the smallest or largest value within 1.5× IQR from the box boundaries. *P < 0.05, **P < 0.01. ***P < 0.001. ****P < 0.0001.

CNV is widely present in the human genome and plays a pivotal role in the occurrence and development of tumors (49). We further explored the association between hypoxia and CNV. **Figures S6C, D** shows the landscape of CNVs in the high and low Hypoxia_DEGs_Score groups. Hypoxia_DEGs_Score showed an excellent positive correlation with the frequency of CNVs of both loss and gain type (**Figures 6A, B**). Further analysis identified 40 differential CNV loci between different hypoxia cohorts. The CNV profiles of the top 20 and all loci are shown in **Figure 6C** and **Table S10**, respectively. We performed an enrichment analysis of possible target genes among 40 significantly different CNV loci (**Figure 6D**). The results suggested that the drug resistance of HCC tumors under hypoxia may be caused by a loss of genome stability, which leads to an increased frequency of CNVs, thereby altering the metabolism of cytochrome P450s and promoting drug resistance.

### General DNA methylation and N6-methyl-adenosine modification of HCC stratified by hypoxic status

Hypoxia can alter DNA methylation status, thereby affecting the transcription and translation of functional proteins, and altering downstream biological behaviors (50). We analyzed differentially methylated positions(DMPs) between high and low Hypoxia_DEGs_Score groups. A total of 1104 DMPs were identified, including 552 upregulated and 552 downregulated DMPs (**Figure S7A**, **Table S11**). The landscape of the top 50 DMPs is shown in **Figure S7B**. According to the enrichment analysis of DMP associated genes, methylation altered bile acid metabolism and possibly also inhibited the p53 signaling pathway, which significantly promoted tumor proliferation (**Figure 6E**). Alterations in the tight junction protein complex affect junction assembly, barrier regulation, and cell polarity, thereby promoting tumor progression and metastasis (51).

The N6-methyladenosine(m6A) DNA modification plays a crucial role in many biological functions, and its dysregulation is associated with cancer progression in HCC (52). We analyzed differences in the transcript levels of 28 m6A regulators (53) between high and low Hypoxia_DEGs_Score groups (**Figure 6F**). Notably, 12 of the 14 m6A readers were significantly different. The reader YTHDF1 promotes tumor progress by influencing ATG2A, ATG14, and HIF-1α (54). At the same time, there were large differences in 7 of the 11 m6A writers. High expression of WTAP promotes tumor progression, by influencing HuR, p21/27, and Ets−1 (55). Interestingly, the expression levels of the FTO and ALKBH3 m6A erasers exhibited a sharp decrease under hypoxia. In general, hypoxia promotes the occurrence and progression of HCC by increasing the abundance of the m6A DNA modification.

### Potential predictive value of Hypoxia_DEGs_Score for immunotherapy response

**Figure 7:**
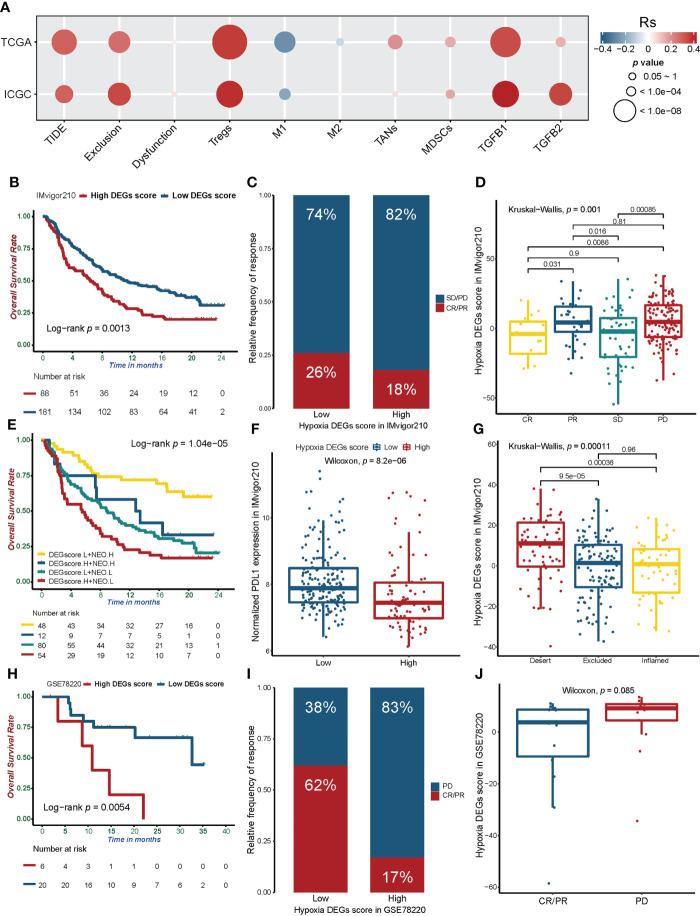
The relationship between the Hypoxia_DEGs_Score and efficacy of immunotherapy. **(A)** Correlation of the Hypoxia_DEGs_Score with the TIDE score, infiltration of immunosuppressive cells, and expression of TGF-β. Rs is the coefficient of rank correlation. **(B, H)** Kaplan-Meier survival curves showing differences in overall survival between the high and low Hypoxia_DEGs_Score groups in the IMviogor210 **(B)** and GSE78220 **(H)** cohorts. **(C, I)** The proportion of patients in the IMvigor210 **(C)** and GSE78220 **(I)** cohorts with different PD-L1 and PD-1 blockade immunotherapy responses. **(D, J)** Difference in the Hypoxia_DEGs_Score between distinct clinical outcomes of anti-PD-L1 and PD-1 treatment in the IMvigor210 **(D)** and GSE78220 **(J)** cohorts. **(E)** Kaplan-Meier survival curves demonstrating distinctions in overall survival stratified by both the Hypoxia_DEGs_Score and tumor neoantigen burden. **(F)** Differences in PD-L1 expression between low and high Hypoxia_DEGs_Score groups in the IMviogor210 cohort. **(G)** Differences in the Hypoxia_DEGs_Score among immune phenotypes, including the inflamed (yellow), excluded (blue), and desert (red) immune types in the IMvigor210 cohort. CR, complete response; PR, partial response; SD, stable disease; PD, progressive disease; NEO, tumor neoantigen burden; L, low; H, high.

Numerous studies have demonstrated that hypoxia can be the core driver of transformation in the tumor immune microenvironment (56). We applied the TIDE method (25) to compare the TME of tumors with different hypoxia statuses (**Tables S5** and **6**). TIDE and Exclusion scores were positively correlated with the Hypoxia_DEGs_Score. Notably, Dysfunction scores were not associated with hypoxia levels (**Figures 7A** and **S8A**).

Further analysis of infiltration by tumor immunosuppressive cells revealed that the Hypoxia_DEGs_Score was significantly positively correlated with infiltration by Tregs, TANs, and MDSCs (**Figures 7A** and **S8B**). High infiltration levels of Tregs lead to attenuated tumor cell killing (57). TANs can promote tumor proliferation and metastasis by releasing elastase (58). The increased abundance of MDSCs in TME increases immune evasion, angiogenesis, and metastasis (59). Patients with high Hypoxia_DEGs_Score had high tumor infiltration of immunosuppressive cells, which promoted tumor progression and resulted in a poor prognosis. M1 macrophages are not only capable of secreting pro-inflammatory mediators, but can kill tumor cells by driving type 1 T helper cell(Th1) responses to produce cytotoxic effects (60). HCC samples with higher Hypoxia_DEGs_Score values had significantly less M1 macrophage infiltration (**Figures 7A** and **S8B**), suggesting an attenuated M1-mediated inflammatory state under hypoxia. TGF-β can increase the number of Tregs, suppress effector T cell activity and attenuate dendritic cell function (61). A positive correlation of the Hypoxia_DEGs_Score with the expression of TGFB1 and TGFB2 (**Figure 7A** and **S8C**) suggests that the hypoxic status of HCC results in a tumor immunosuppressive state with increased infiltration of immunosuppressive cells.

Immunotherapy based on PD-1 and PD-L1 blockade has dramatically improved the prognosis of patients. Considering that the Hypoxia_DEGs_Score can predict the TME phenotype of HCC, we selected an anti-PD-L1 treated cohort(IMvigor210) (20) and an anti-PD-1 treated cohort(GSE78220) (21) for further analysis. The results showed that patients with low Hypoxia_DEGs_Score values exhibited significant clinical benefits and markedly prolonged overall survival compared with those with a high Hypoxia_DEGs_Score (**Figures 7B–D**). In the IMvigor210 cohort, we found that the Hypoxia_DEGs_Score of the patients in the complete response (CR) group was lower than that of other groups, and the scores in the progressive disease(PD) group were higher than in the other groups (**Figure 7D**). Further analysis revealed that patients with high Hypoxia_DEGs_Score values and a low tumor neoantigen burden had the poorest prognosis (**Figure 7E**). PD-L1 expression was significantly elevated in patients with low Hypoxia_DEGs_Score values, suggesting a potential benefit of anti-PD-1/L1 immunotherapy (**Figure 7F**). In the Hypoxia_DEGs_Score analysis of different immune subtypes, we found that the immune desert type had higher scores than the immune excluded and inflammatory types (**Figure 7G**). Consequently, the analysis indicates that the Hypoxia_DEGs_Score model can guide ICIs drug selection and predict the efficacy of anti-PD-1/PD-L1 immunotherapy.

### The Hypoxia_DEGs_Score model predicts drug responses

To further investigate the association between drug resistance and hypoxic stress, we assessed the correlation between the Hypoxia_DEGs_Score and drug responses in various cancer cell lines based on the GDSC database (35). The results revealed a significant correlation between the responses to 39 drugs and the Hypoxia_DEGs_Score (**Figure S9A**, **Table S12**). Among them, the sensitivity of the tumors to 14 drugs increased as hypoxia progressed, and conversely, tumor resistance to 25 drugs increased as hypoxia progressed. In addition, drug sensitivity in the high Hypoxia_DEGs_Score tumors was mainly associated with drugs targeting the EGFR signaling pathway. By contrast, drug tolerance was primarily associated with drugs targeting the PI3K/Akt and histone acetylation signaling pathways (**Figure S9B**, **Table S12**).

**Figure 8:**
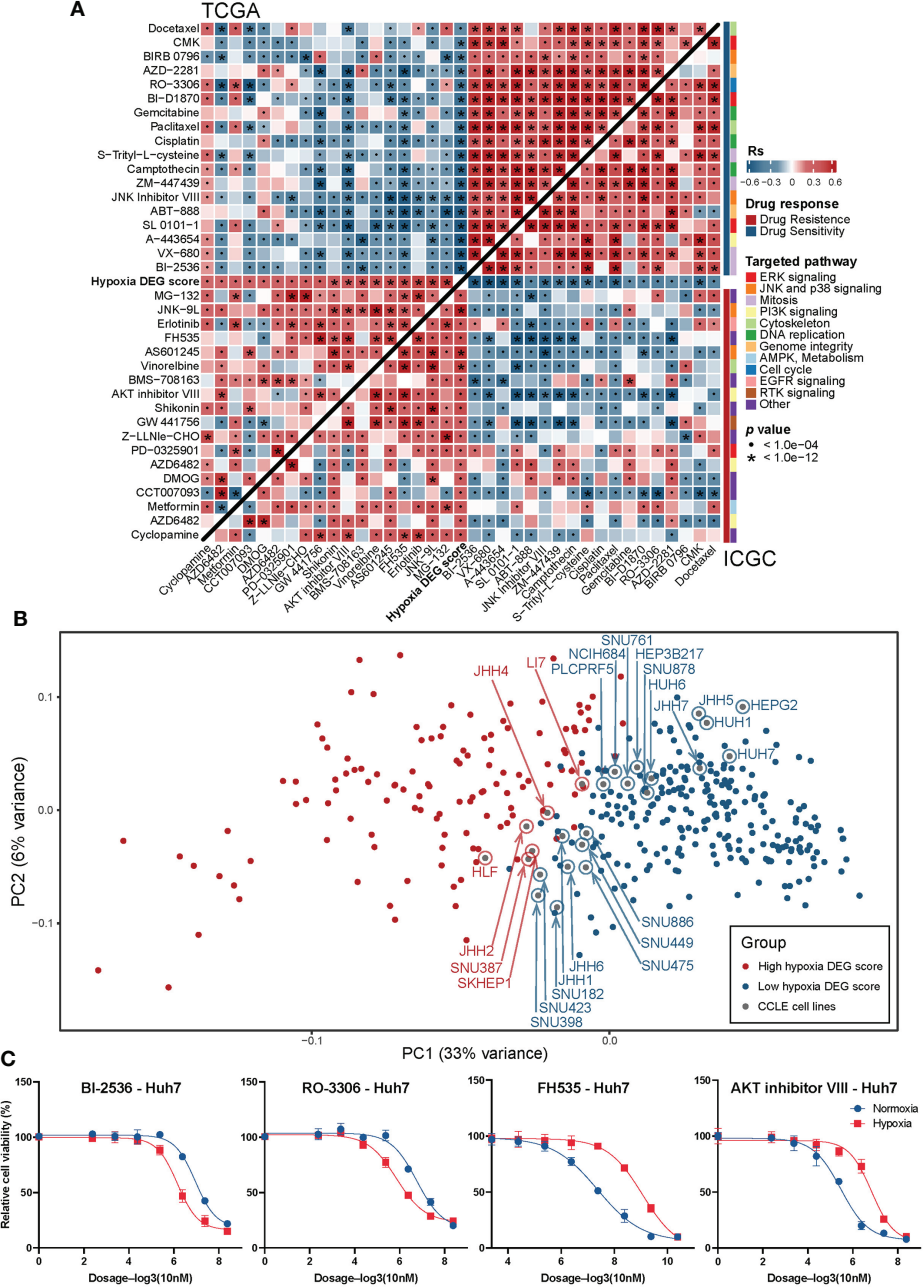
Relationship between the Hypoxia_DEGs_Score and drug response. **(A)** Correlation between the Hypoxia_DEGs_Score and drug sensitivity assessed using Spearman correlation analysis in the TCGA-LIHC and ICGCLIRI-JP cohort. The upper left triangle shows the analysis of the TCGA-LIHC cohort; the lower right triangle shows the analysis of the ICGC-LIRI-JP cohort. Red indicates ineffective drugs (tumor is resistant), and blue indicates effective drugs (tumor is sensitive). Rs is the coefficient of rank correlation. Each small rectangle on the right represent the targeted pathways corresponding to the drugs. *P < 1.0e-04; •P < 1.0e-12 **(B)** Principal component analysis for the transcriptome profiles of 251 hypoxia-related DEGs based on the combination between HCC cell lines and patient samples from the GDSC dataset and the TCGA-LIHC cohort. Red represents HCC samples and cell lines with high Hypoxia_DEGs_Score values; blue Represents low Hypoxia_DEGs_Score. **(C)** Dose-response curves for the the mean value of cell viability for BI-2536, RO-3306, FH535, and Akt inhibitor-VIII in the HCC cell lines Huh7 **(C)** and HepG2 (**Figure S12C**) incubated under hypoxic (red, n = 4) and normoxic (blue, n = 4) conditions. Cell viability was normalized to that of cells mock-treated with dimethyl sulfoxide (vehicle control). The error bars indicate the mean ± SD. The sigmoidal dose-response curves were fitted to the data.

For HCC, we applied a ridge regression model to analyze the correlation of Hypoxia_DEGs_Score with the drug response based on the TCGA-LIHC and ICGC-LIRIJP datasets (**Figure 8A**, **Table S13**). Further analysis identified 20 drugs that were effective and 20 drugs to which the tumors were resistant under hypoxia. In hypoxic HCC, most effective drugs were found to target cell cycle-related signaling pathways. By contrast, hypoxic HCC appears to be resistant to most drugs targeting the PI3K/Akt signaling pathway. These results were consistent with the analysis of various cancer types.

To directly confirm the association between the Hypoxia_DEGs_Score and the drug response, we conducted a PCA analysis. The results showed that HepG2, Huh7, and Huh1 were the HCC cell lines with the lowest hypoxia levels, while HLF, JHH2, and SNU387 had the highest hypoxia levels (**Figure 8B**). Additionally, we cultured HepG2 and Huh7 cells under hypoxic and normoxic conditions, and treated them with representative drugs. The results indicated that under hypoxic conditions, HCC exhibited higher drug sensitivity to BI-2536 and RO-3306 but higher resistance to FH535 and AKT inhibitor VIII (**Figures 8C** and **S9C**). These results were consistent with our computational prediction. Thus, the Hypoxia_DEGs_Score is a potential biomarker for establishing appropriate therapeutic regimens.

## Discussion

Due to differences in organ function, metabolic background, and blood supply, the hypoxia status varies significantly in different tumors. As a heterogeneous disease, HCC is subject to comprehensive and diverse biological processes, resulting in a variable and inconsistent prognosis. The existing biomarkers used to guide diagnosis and treatment cannot fully meet the clinical needs. Therefore, establishing new biomarkers with higher predictive value is critical for maximizing the clinical benefits of a personalized regimen and accurate prognostic assessment. We distinguished three HCC hypoxia phenotypes based on 15 hypoxia signature genes and constructed the HCC-specific hypoxia scoring model Hypoxia_DEGs_Score. The robustness of the Hypoxia_DEGs_Score model was validated in four independent HCC cohorts from different sources (**Figures 4C–K**, **S5A–C**, and **Table S1**). Encouragingly, the Zhongshan and Tongji cohorts used as validation datasets also had a prominent area under curve (AUC) of the ROC, and patients with low Hypoxia_DEGs_Score values had a significantly better prognosis.

Hypoxia is a powerful driving force that promotes the heterogenization and genomic evolution of cancer. However, the understanding its association with specific mutational processes and CNVs remains limited (62). In our analysis, the mutation frequency of tumor driver genes generally increased under hypoxia (**Figure 5A**), and the frequency of CNVs also surged with rising Hypoxia_DEGs_Score values (**Figures 6A, B**). These results are consistent with the widespread elevation of the mutational burden across all mutational types related to hypoxia in pan-cancer studies (62). TP53 mutations prevent apoptosis and lead tumors into the dynamic cycle of hypoxia (63, 64). The high mutation rate of TP53 in the high hypoxia group indicated that TP53 mutations play a pivotal role in the development of HCC under hypoxia (**Figures 5B, C**). It has been reported that SETD2 mutations were more frequently found in the hypoxic group of kidney tumors (65, 66), and the hypoxic group of HCC also had more patients with mutant SETD2 in our study (**Figures 5B, C**). It is worth noting that patients with both mutant SETD2 and a hyper-hypoxic status had the worst prognoses (**Figures 5E, F**). An enriched drug metabolism-cytochrome P450 gene set based on the target genes of hypoxic differential CNV loci indicated that CNVs of cytochrome P450 genes may enhance drug resistance in hypoxic HCC, in agreement with previous literature (67). In our study, the PPAR signaling pathway was enriched in three analyses, respectively in the GSVA of high and low clustering groups, 251 DEG enrichment analysis, and CNV loci-related genes enrichment analysis (**Figures 2A**, **4B**, and **6D**). Downregulation of the PPAR signaling pathway has been reported to promote tumor growth and the EMT resulting in metastasis. Therefore, CNVs may promote the tumor growth and metastasis of HCC by downregulating the PPAR signaling pathway under hypoxia.

Aberrant methylation of genes affects multiple biological functions of tumors, such as cell cycle, DNA repair, toxin catabolism, cell adherence, apoptosis, and angiogenesis (68). In our study, hypoxia inhibited or promoted the expression of various genes through methylation modification of DNA, including genes related to cholesterol metabolism, p53 signaling, tight junction protein function and ATP-binding cassette (ABC) transporters, thereby altering tumor metabolism, cell cycle, DNA repair, cell adherence, and drug catabolism (**Figure 6E**). Previous reports indicate that increased expression of ABCA1 and ABCC1 is positively correlated with chemotherapy resistance (69–72). In our analysis, ABCA1 and ABCC1 were methylated under hypoxia, suggesting that hypoxia increases the number of ABC transporters and promotes drug resistance in HCC by downregulating the methylation of ABCA1 and ABCC1.

Hypoxia is an essential feature of the TME and is closely linked to cell proliferation, angiogenesis, metabolism, and tumor immunity (73). Our study showed that a high Hypoxia_DEGs_Score was positively correlated with the TIDE score (**Figures 7A** and **S8A**). A high TIDE score indicates that the patient’s tumor is immunosuppressed, which predicts a poor response to ICIs treatment (25). The positive correlation between the Hypoxia_DEGs_Score and the Exclusion score indicated that hypoxic immunosuppression in HCC was dominated by immune cell reduction rather than immune dysfunction (**Figures 7A** and **S8A**). The increased infiltration of TAN, MDSC, and Treg cells indicates the suppression of immune function and promotion of tumor progression in HCC under hypoxia (**Figures 7A** and **S8B**). Furthermore, TGFB1 and TGFB2 were positively correlated with the Hypoxia_DEGs_Score, further illustrating the immunosuppressive state induced by hypoxia. We evaluated the predictive power of Hypoxia_DEGs_Score for responses to anti-PD-L1 and anti-PD-1 immunotherapy. Although the data for these assessments were not derive from HCC cohorts, the results showed that predicting survival time and treatment response was significantly associated with the Hypoxia_DEGs_Score (**Figures 7B–J**). Consistently, patients with high scores had a poor prognosis in four independent cohorts (**Figures 4C–K**, **S5A–C**). At the same time, the immunotherapy outcome was also compatible with the TIDE score and TME status of HCC under aggravated hypoxia (**Figures 7A** and **S8**). These results indicated that patients with high Hypoxia_DEGs_Score values have a poorer prognosis and may not benefit from ICIs therapy.

Hypoxia promotes drug resistance through apoptosis, autophagy, DNA damage, mitochondrial activity, p53, drug efflux, and an acidic microenvironment (56). Therefore, it is vital to find a regime that can effectively eliminate tumors in a hypoxic environment. The pan-cancer analysis was not limited to HCC, so we analyzed the drug response of HCC under hypoxic stress based on TCGA-LIHC and ICGC-LIRI-JP. The primary targeted pathway of effective drugs was cell cycle-related, and the main targeted pathway of drugs to which the tumors were resistant was PI3K/Akt signalling (**Figure 8A**). To confirm these findings, we placed HCC cell lines in hypoxic and normoxic environments to assess their drug sensitivity (**Figures 8C** and **S9C**), and the results were consistent with the predictions. Notably, the response to AKT inhibitor VIII and RO-3306, which respectively target the PI3K/mTOR and cell cycle pathways, was consistent with the pan-cancer drug prediction results, but FH535 and BI-2536 did not appear in the results. These analyses suggest that patients with higher Hypoxia_DEGs_Score values may benefit from drugs against cell cycle signaling pathways rather than those inhibiting the PI3K/mTOR pathway. It is suggested that Hypoxia_DEGs_Score could serve as a potential biomarker to guide drug selection for patients.

The advancement of sequencing technology and understanding of the tumor microenvironment, as well as the combination of molecular and genetic features, and clinicopathological factors, offer new hope for precise prediction and personalized therapy. Therefore, genetic signatures bridging hypoxia and prognosis offer promising guidelines for exploring and treating HCC. Our study provides new possibilities for improving therapy outcomes in hepatocellular carcinoma by identifying distinguishing hypoxic states and enabling personalized regimes.

In conclusion, we performed a systematic, comprehensive analysis of the effects of hypoxia on multiple HCC cohorts based on the hypoxia signature. The Hypoxia_DEGs_Score prediction model was constructed, and exhibited an excellent survival prediction value. It was revealed that hypoxia not only has a complex effect on the TME, but also has an extensive impact on genome instability and DNA methylation. A comprehensive evaluation of the hypoxia status of individual HCC cases will help us deepen our understanding of the altered biological functions of HCC and reveal the key role of hypoxia in targeted therapy and immunotherapy. Hypoxia_DEGs_Score can greatly aid the development of optimal personalized immunotherapy regimens for HCC patients, with the potential to greatly improve the prognosis.

## Data availability statement

The Tongji cohort's RNA sequencing data presented in the study are deposited in the Gene Expression Omnibus repository (https://www.ncbi.nlm.nih.gov/geo/), accession number GSE201868. The other RNA sequencing, Whole Genome Sequencing, methylation chip, and clinicopathological data of HCC patients, or patients with immune checkpoint were described in method section “Data collection and processing” and Supplementary Material. The resources, tools and codes used in our analyses were described in each method section in the methods.

## Author contributions

GC, JZ and DN designed this study. YZ, YX, JL, and GL extracted the information from the databases and analyzed the data. GC completed the verification experiments. CY, XC, HL, and ZD supervised the entire study. GC wrote the manuscript. All authors revised the manuscript.

## Funding

This work was supported by the National Nature Science Foundation of China (No’s. 81874065 and 81874149), National Basic Research Program of China (2020YFA0710700), Tongji Hospital (HUST) Fundation for Excellent Young Scientist (No. 2020YQ05), the first level of the public health youth top talent project of Hubei province.

## Acknowledgments

The authors would like to sincerely thank the TCGA, ICGC, Zhongshan hosipital, GEO, IMvigor, and GDSC for data sharing, as well as TIDE, cibersort, and, cBioPortal for providing data processing. In particular, the author Xiaoping Chen would like to express deepest thanks to Dr. Ivan Hajal for his support in revising this manuscript.

## Conflict of interest

ZD served as a speaker and consultant for Bayer, Eisai, Roche, MSD, Astra-Zeneca, Innovent, Hengrui, and BeiGene. XC served as a consultant of Bayer, Eisai, and Hengrui. XC conducts studies for Hengrui and CTTQ.

The remaining authors declare that the research was conducted in the absence of any commercial or financial relationships that could be constructed as a potential conflict of interest.

## Publisher’s note

All claims expressed in this article are solely those of the authors and do not necessarily represent those of their affiliated organizations, or those of the publisher, the editors and the reviewers. Any product that may be evaluated in this article, or claim that may be made by its manufacturer, is not guaranteed or endorsed by the publisher.

## Supplementary material

Supplementary Figure 1 Overview of study design. Flowchart of the steps in the performed analyses.

Supplementary Figure 2 Unsupervised clustering and expression of 15 hypoxia signature genes. **(A)** Unsupervised clustering analysis of the 15 genes hypoxia signature with the comparison of clinicopathological features between three distinct clusters for ICGC-LIRI-JP. **(B, C)** The expression of 15 hypoxia signature genes across distinct hypoxia groups and normal tissue group in TCGA-LIHC **(B)** and ICGC-LIRI-JP **(C)** cohorts. *, P< 0.05; **, P< 0.01; ***, P< 0.001; ****, P< 0.0001.

Supplementary Figure 3 Correlation analysis and mutation status of 15 hypoxia signature genes. **(A)** Heatmap displays the correlation between 15 hypoxia signature genes and hypoxia-related signatures. Exhibited values represent correlation coefficients and were satisfied with FDR< 0.05. **(B)** The mutation frequency of 15 hypoxia signature genes in 364 patients with HCC from TCGA-LIHC cohort. Each column represented individual patients. **(C)** The expression of 15 hypoxia signature genes between normal, gain, and loss of CNV. *, P < 0.05; **, P < 0.01; ***, P < 0.001; ****, P < 0.0001.

Supplementary Figure 4 Biological characteristics and immune cell infiltration of distinct hypoxia groups. **(A)** Heatmap visualizing the GSVA enrichment analysis based on GO terms shows the activation states of biological pathways in hypoxia high and low groups. **(B)** The infiltration of 22 immune cells between distinct hypoxia groups in TCGA-LIHC cohorts. * P < 0.05; ** P < 0.01; *** P < 0.001; **** P < 0.0001. Correlation analysis of Hypoxia_DEGs_Score with HIF1A transcript levels in TCGA **(C)** and ICGC cohorts **(D)**.

Supplementary Figure 5 Survival status in the different hypoxic statuses and multivariate Cox regression analysis and comprehensive nomogram for the Hypoxia_DEGs_Score. **(A–C)** Scatter plots of survival status and risk curves exhibited prognosis and Hypoxia_DEGs_Score in patients from TCGA-LIHC **(A)**, ICGC-LIRI-JP **(B)**, and Zhongshan **(C)** cohorts. **(D)** Forest plots showing multivariate Cox regression model analysis in the TCGA-LIHC and ICGC-LIRI-JP cohorts. The horizontal line represents the 95% confidence interval (CI) for each group. The vertical dotted line represents all patients’ hazard ratios (HR). **(E)** The comprehensive nomogram predicting the clinical outcomes of HCC patients with 2- and 3-year OS based on the TCGA-LIHC cohort.

Supplementary Figure 6 Calibration of comprehensive nomogram and landscape of CNV of different hypoxic statuses. **(A, B)** Calibration plots for predicting the 2-year **(A)** and 3-year **(B)** OS of HCC patients in the TCGA-LIHC cohort. Nomogram-predicted probability of survival was plotted on the x-axis, and actual survival was plotted on the y-axis. **(C, D)** The landscape of CNV in high **(A)** and low **(B)** Hypoxia_DEGs_Score groups.

Supplementary Figure 7 The beta value of DNA methylation differential sites in the different hypoxic statuses. **(A)** Heatmap exhibiting the beta value of 1104 DNA methylation differential sites in high and low Hyposia_DEGs_Score groups. **(B)** Heatmap displayed the beta value of top 50 DNA methylation differential sites in high and low Hyposia_DEGs_Score groups.

Supplementary Figure 8 Changes of the tumor immune microenvironment in groups with different hypoxic statuses. **(A–C)** Scatterplots showing the correlation of the Hypoxia_DEGs_Score with the TIDE score **(A)**, infiltration of immunosuppressive cells **(B)**, and expression of TGF-β **(C)** according to Spearman’s rank correlation analysis in the TCGA-LIHC and ICGCLIRI-JP cohorts. Rs is the coefficient of rank correlation.

Supplementary Figure 9 Relationship between the Hypoxia_DEGs_Score and drug response. **(A)** Correlation between the Hypoxia_DEGs_Score and drug sensitivity based on Spearman correlation analysis in GDSC. Each column represents a drug. The brightness of the column indicates the significance of the correlation. Rs is the coefficient of rank correlation. The height of the column indicates the correlation between Hypoxia_DEGs_Score and drug resistance (Rs > 0) or drug sensitivity (Rs< 0). **(B)** Signaling pathways targeted by ineffective (red) or effective (blue) drugs according to the Hypoxia_DEGs_Score. Drug names were listed on the horizontal axis and the signaling pathway targeted by the drug on the vertical axis. The bar graph on the right shows the number of drugs targeting each signaling pathway. **(C)** Dose-response curves for the the mean value of cell viability for BI-2536, RO-3306, FH535, and Akt inhibitor-VIII in the HCC cell lines HepG2 under hypoxic (red, n = 4) and normoxic (blue, n = 4) conditions.
