# Supplementary figures and images for: A Novel hepatocellular carcinoma specific hypoxic related signature for predicting prognosis and therapeutic responses
Source: Front Immunol. 2022 Aug 17;13:997316. doi: 10.3389/fimmu.2022.997316 (PMC9428591; doi:10.3389/fimmu.2022.997316)

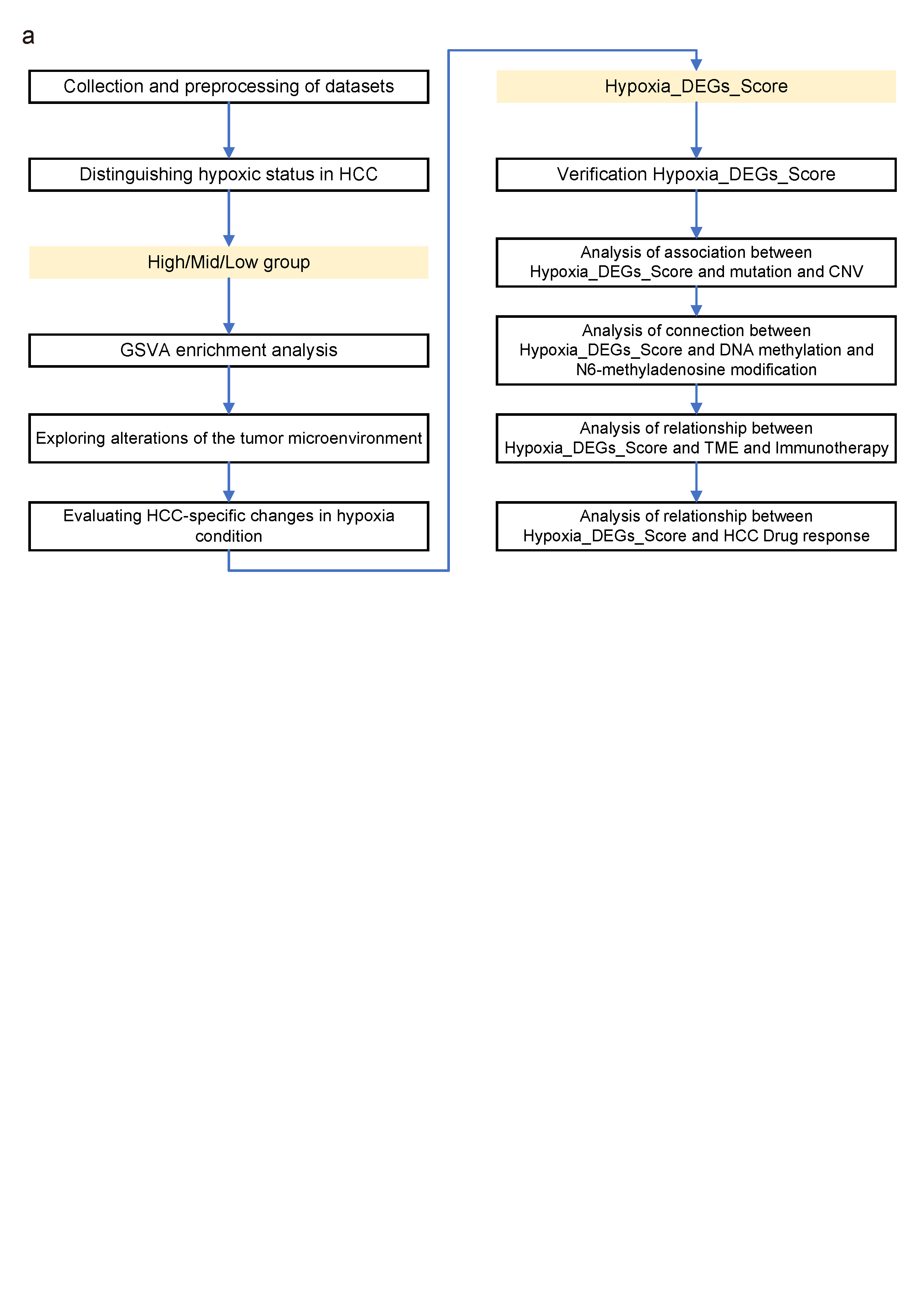

Supplement: Supplementary Figure 1 — Overview of study design. Flowchart of the steps in the performed analyses. [file Image_1.jpeg]

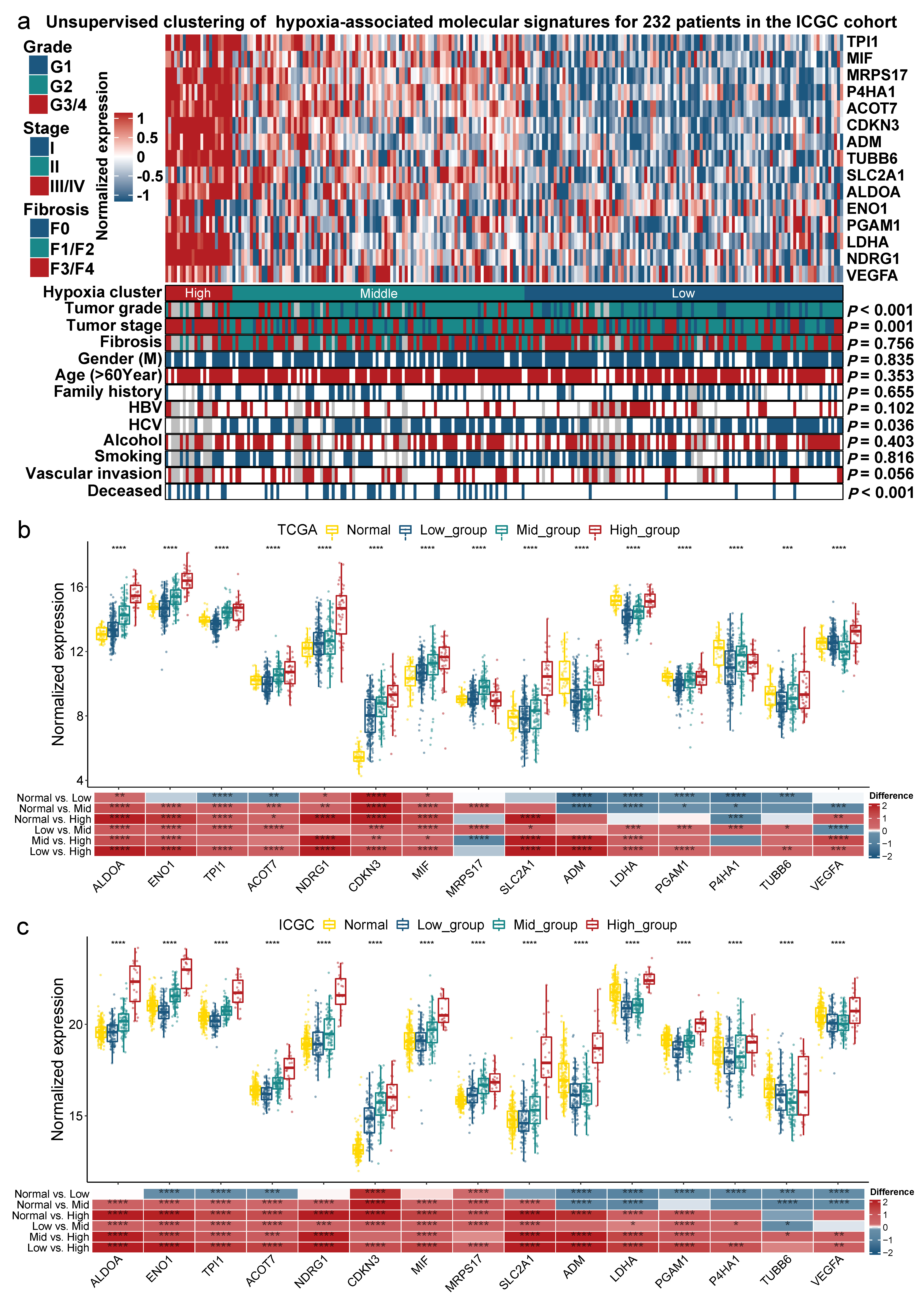

Supplement: Supplementary Figure 2 — Unsupervised clustering and expression of 15 hypoxia signature genes. (A) Unsupervised clustering analysis of the 15 genes hypoxia signature with the comparison of clinicopathological features between three distinct clusters for ICGC-LIRI-JP. (B, C) The expression of 15 hypoxia signature genes across distinct hypoxia groups and normal tissue group in TCGA-LIHC (B) and ICGC-LIRI-JP (C) cohorts. *, P< 0.05; **, P< 0.01; ***, P< 0.001; ****, P< 0.0001. [file Image_2.jpeg]

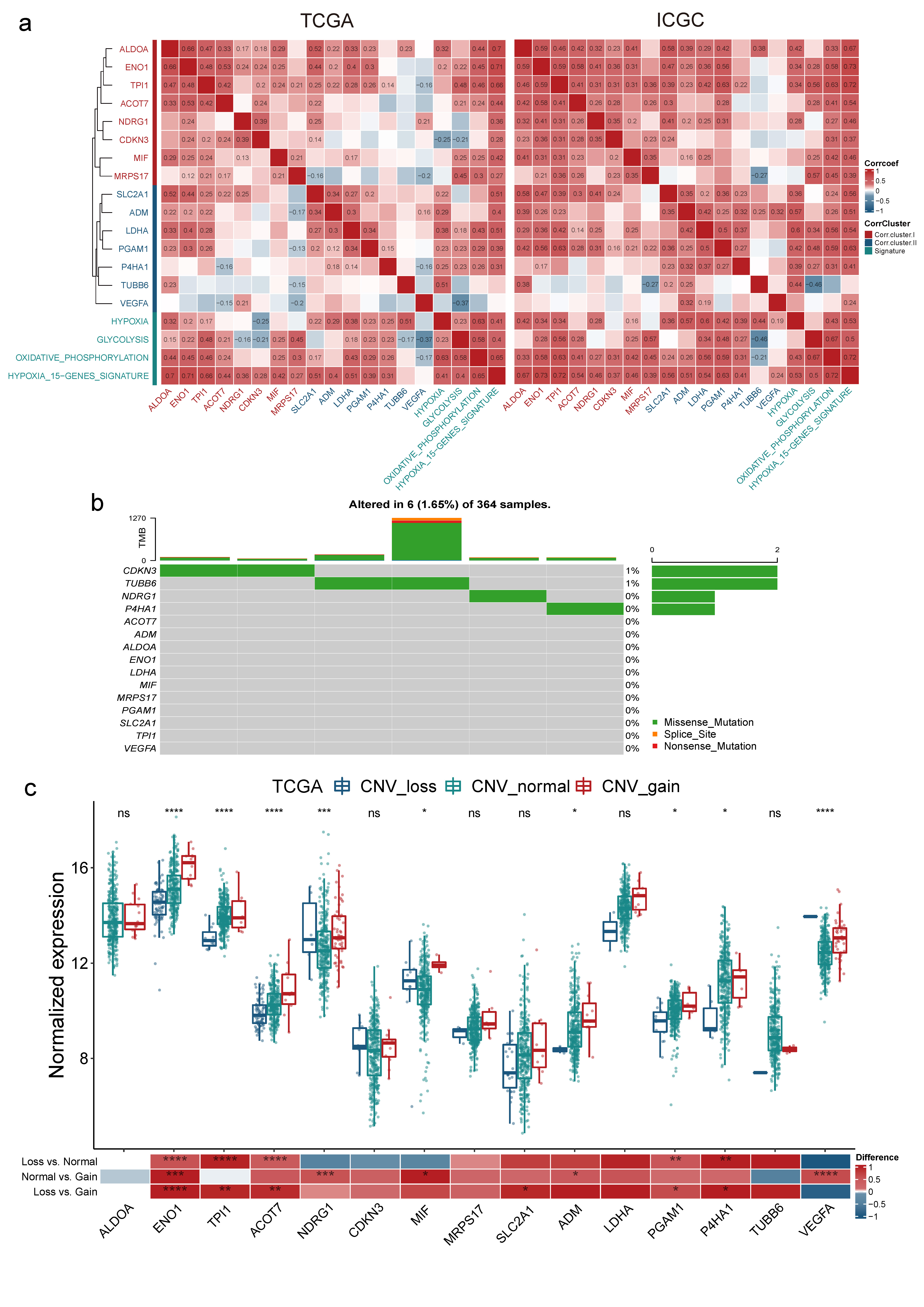

Supplement: Supplementary Figure 3 — Correlation analysis and mutation status of 15 hypoxia signature genes. (A) Heatmap displays the correlation between 15 hypoxia signature genes and hypoxia-related signatures. Exhibited values represent correlation coefficients and were satisfied with FDR< 0.05. (B) The mutation frequency of 15 hypoxia signature genes in 364 patients with HCC from TCGA-LIHC cohort. Each column represented individual patients. (C) The expression of 15 hypoxia signature genes between normal, gain, and loss of CNV. *, P < 0.05; **, P < 0.01; ***, P < 0.001; ****, P < 0.0001. [file Image_3.jpeg]

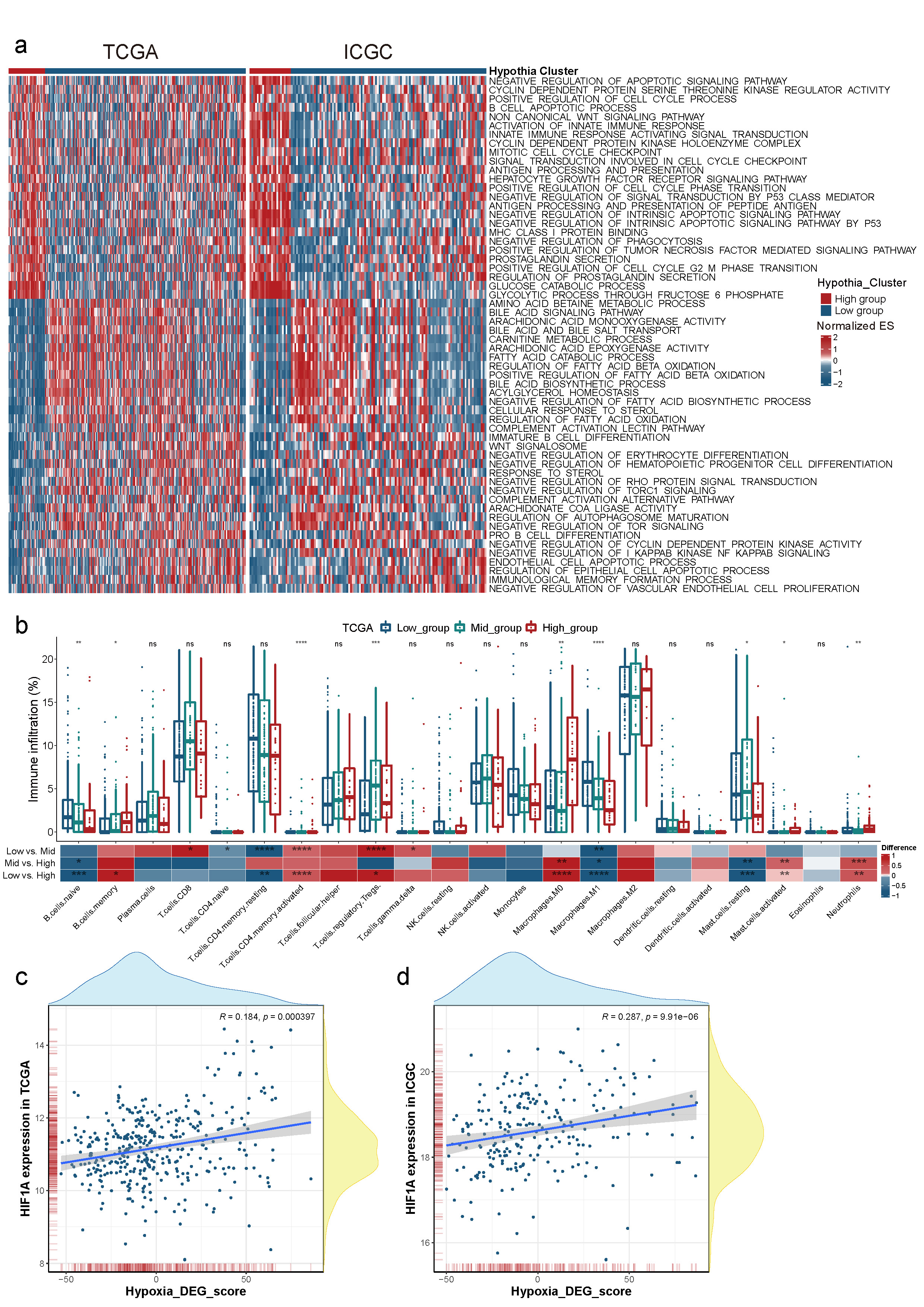

Supplement: Supplementary Figure 4 — Biological characteristics and immune cell infiltration of distinct hypoxia groups. (A) Heatmap visualizing the GSVA enrichment analysis based on GO terms shows the activation states of biological pathways in hypoxia high and low groups. (B) The infiltration of 22 immune cells between distinct hypoxia groups in TCGA-LIHC cohorts. * P < 0.05; ** P < 0.01; *** P < 0.001; **** P < 0.0001. Correlation analysis of Hypoxia_DEGs_Score with HIF1A transcript levels in TCGA (C) and ICGC cohorts (D). [file Image_4.jpeg]

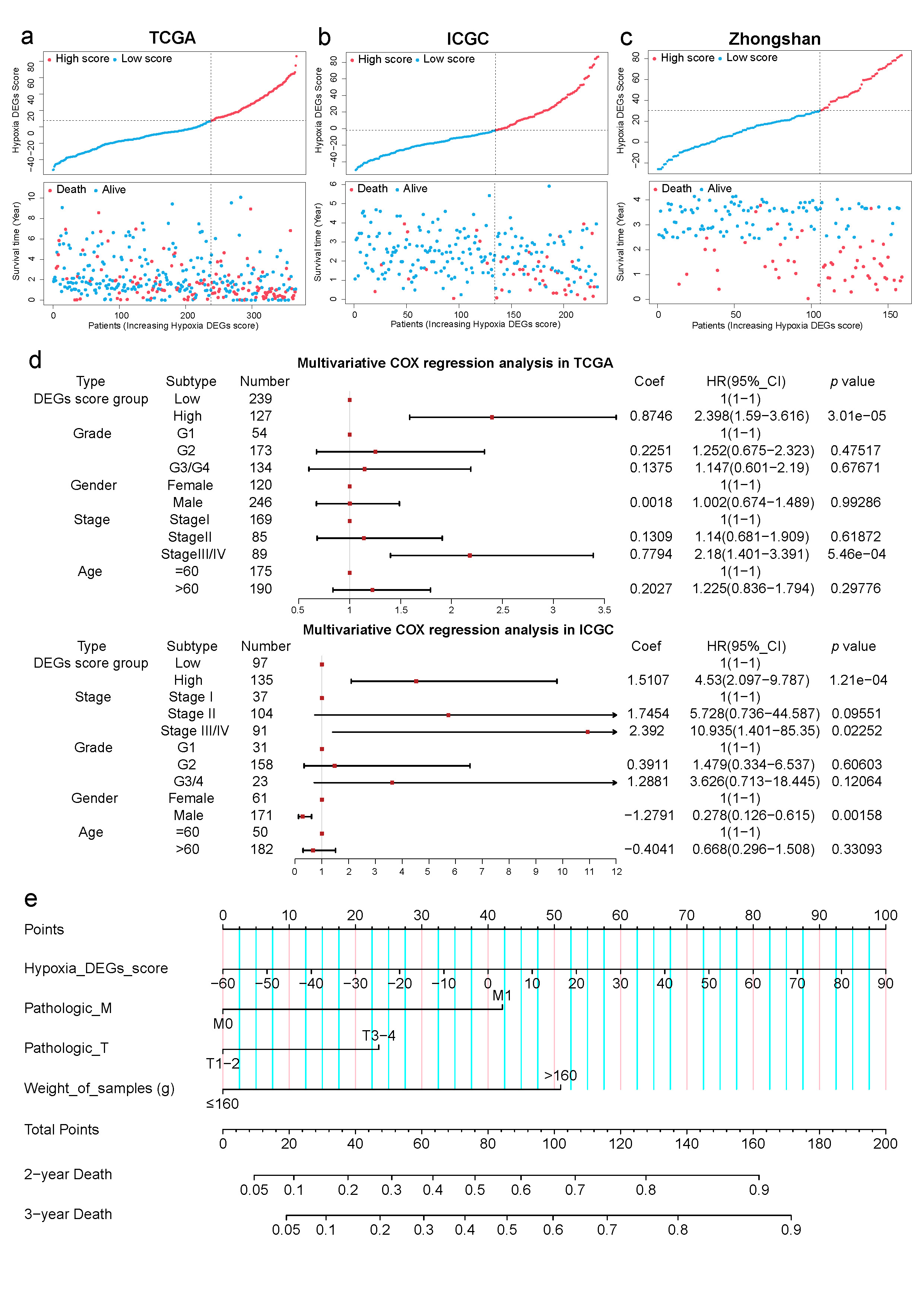

Supplement: Supplementary Figure 5 — Survival status in the different hypoxic statuses and multivariate Cox regression analysis and comprehensive nomogram for the Hypoxia_DEGs_Score. (A–C) Scatter plots of survival status and risk curves exhibited prognosis and Hypoxia_DEGs_Score in patients from TCGA-LIHC (A), ICGC-LIRI-JP (B), and Zhongshan (C) cohorts. (D) Forest plots showing multivariate Cox regression model analysis in the TCGA-LIHC and ICGC-LIRI-JP cohorts. The horizontal line represents the 95% confidence interval (CI) for each group. The vertical dotted line represents all patients’ hazard ratios (HR). (E) The comprehensive nomogram predicting the clinical outcomes of HCC patients with 2- and 3-year OS based on the TCGA-LIHC cohort. [file Image_5.jpeg]

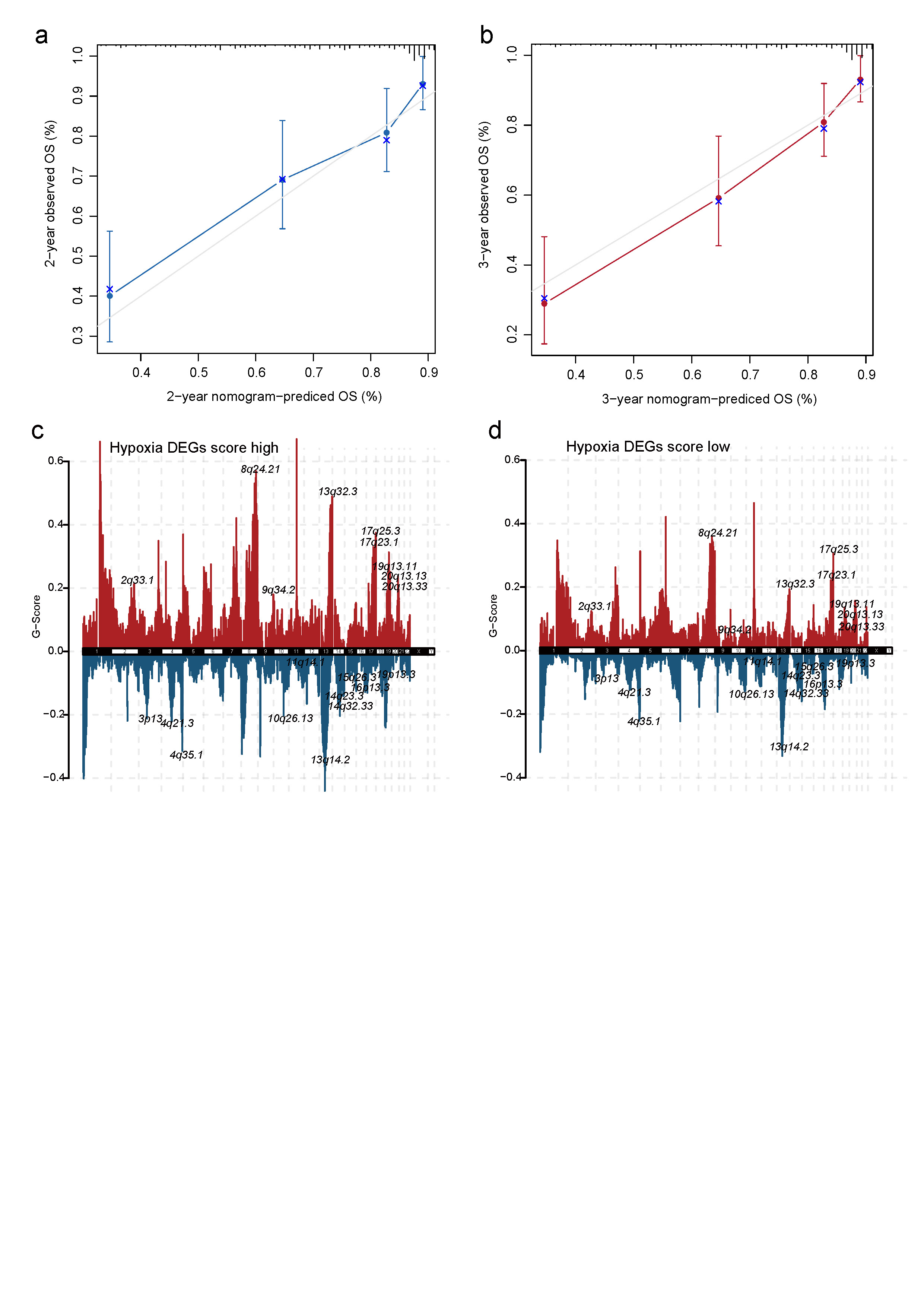

Supplement: Supplementary Figure 6 — Calibration of comprehensive nomogram and landscape of CNV of different hypoxic statuses. (A, B) Calibration plots for predicting the 2-year (A) and 3-year (B) OS of HCC patients in the TCGA-LIHC cohort. Nomogram-predicted probability of survival was plotted on the x-axis, and actual survival was plotted on the y-axis. (C, D) The landscape of CNV in high (A) and low (B) Hypoxia_DEGs_Score groups. [file Image_6.jpeg]

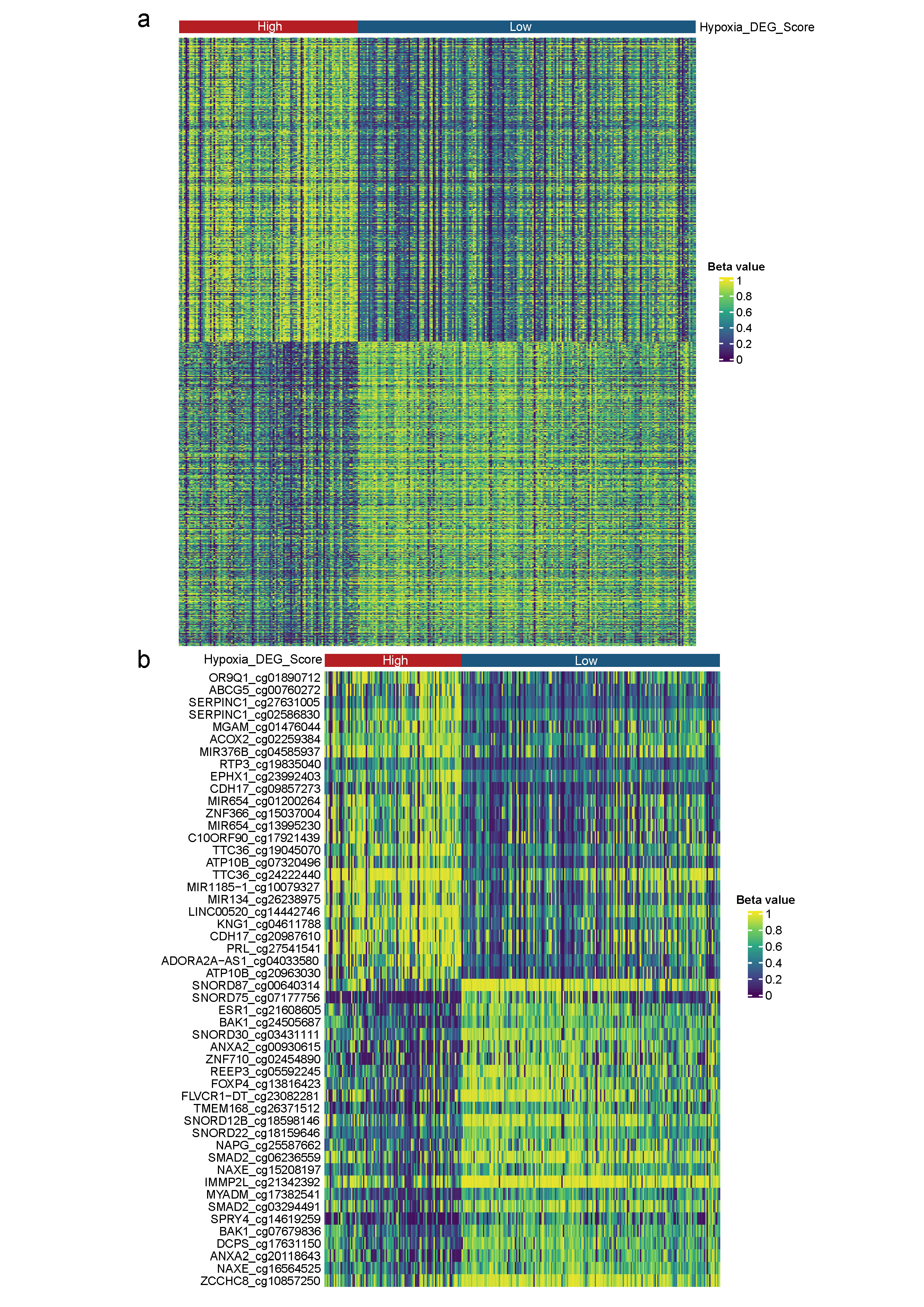

Supplement: Supplementary Figure 7 — The beta value of DNA methylation differential sites in the different hypoxic statuses. (A) Heatmap exhibiting the beta value of 1104 DNA methylation differential sites in high and low Hyposia_DEGs_Score groups. (B) Heatmap displayed the beta value of top 50 DNA methylation differential sites in high and low Hyposia_DEGs_Score groups. [file Image_7.jpeg]

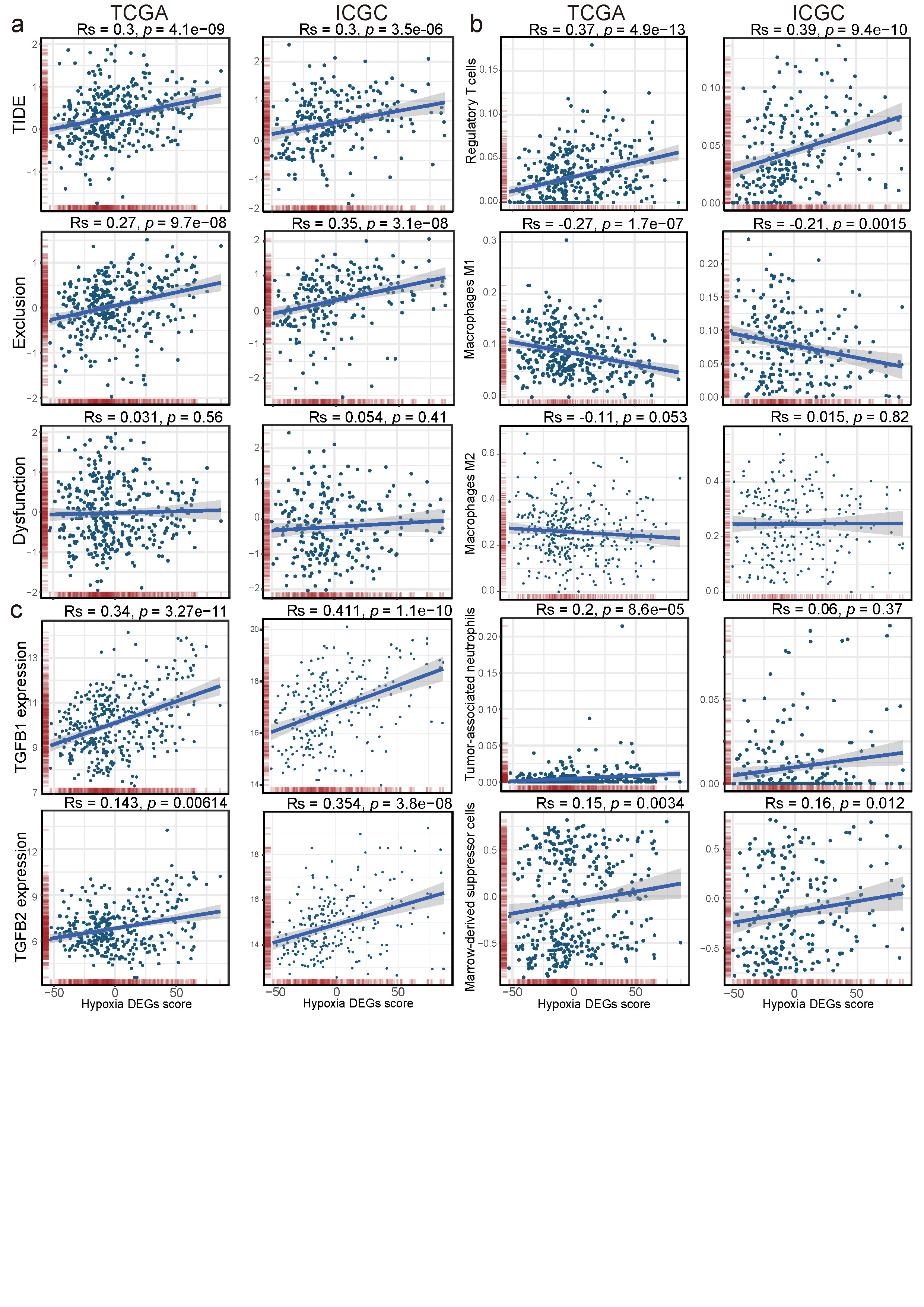

Supplement: Supplementary Figure 8 — Changes of the tumor immune microenvironment in groups with different hypoxic statuses. (A–C) Scatterplots showing the correlation of the Hypoxia_DEGs_Score with the TIDE score (A), infiltration of immunosuppressive cells (B), and expression of TGF-β (C) according to Spearman’s rank correlation analysis in the TCGA-LIHC and ICGCLIRI-JP cohorts. Rs is the coefficient of rank correlation. [file Image_8.jpeg]

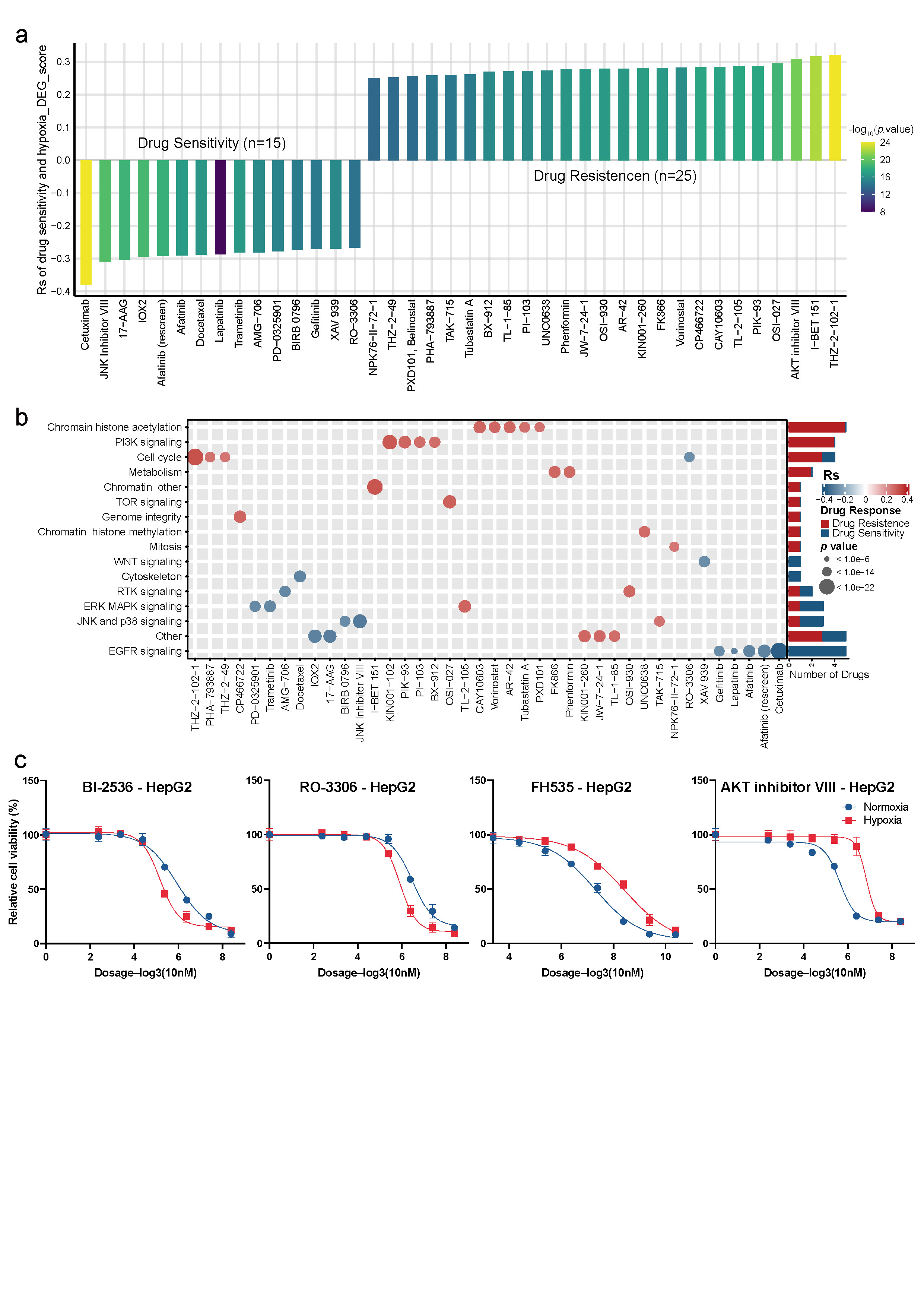

Supplement: Supplementary Figure 9 — Relationship between the Hypoxia_DEGs_Score and drug response. (A) Correlation between the Hypoxia_DEGs_Score and drug sensitivity based on Spearman correlation analysis in GDSC. Each column represents a drug. The brightness of the column indicates the significance of the correlation. Rs is the coefficient of rank correlation. The height of the column indicates the correlation between Hypoxia_DEGs_Score and drug resistance (Rs > 0) or drug sensitivity (Rs< 0). (B) Signaling pathways targeted by ineffective (red) or effective (blue) drugs according to the Hypoxia_DEGs_Score. Drug names were listed on the horizontal axis and the signaling pathway targeted by the drug on the vertical axis. The bar graph on the right shows the number of drugs targeting each signaling pathway. (C) Dose-response curves for the the mean value of cell viability for BI-2536, RO-3306, FH535, and Akt inhibitor-VIII in the HCC cell lines HepG2 under hypoxic (red, n = 4) and normoxic (blue, n = 4) conditions. [file Image_9.jpeg]
